# Toward Practical Li‐Ion Cells With Li/Mn‐Rich Layered Oxide Cathodes: A Techno‐Economic Perspective on Material and Cell Design

**DOI:** 10.1002/advs.202512467

**Published:** 2025-09-30

**Authors:** Anindityo Arifiadi, Sebastian Oster, Donggun Eum, Dominik Voigt, Andrzej Kulka, Hyuck Hur, Martin Winter, Johannes Kasnatscheew

**Affiliations:** ^1^ MEET Battery Research Center Institute of Physical Chemistry University of Münster Corrensstr. 46 48149 Münster Germany; ^2^ International Graduate School for Battery Chemistry Characterization Analysis Recycling and Application (BACCARA) University of Münster Corrensstr. 40 48149 Münster Germany; ^3^ Department of Materials Science and Engineering Stanford University Stanford CA 94305 USA; ^4^ Faculty of Energy and Fuels AGH University of Science and Technology Al. Mickiewicza 30 Krakow 30‐059 Poland; ^5^ Advanced Cell Research Center LG Energy Solution Daejon 34122 South Korea; ^6^ Helmholtz Institute Münster IMD‐4 Forschungszentrum Jülich GmbH Corrensstr. 46 48149 Münster Germany

**Keywords:** cell design, composition tuning, coating, crystal engineering, doping, electrolytes, lithium ion batteries, Li/Mn‐rich layered oxide, oxygen redox, single crystal, techno‐economic analysis

## Abstract

Li/Mn‐rich layered oxide (LMR) cathode active materials offer a pathway towards high specific energy and low‐cost Li ion batteries (LIBs) due to their high practical specific discharge capacity (>250 mAh g^−1^) at moderate discharge voltages (≈3.5 V). However, oxygen redox requires electrochemical activation at high cathode potentials (> 4.5 V vs Li|Li^+^), resulting in bulk degradation and surface reactivity. This perspective first summarizes the literature‐known efforts to elucidate the oxygen redox mechanism and then proposes strategies for systematic R&D of LMR, supported with techno‐economic analysis. Initially, bulk degradation should be addressed via compositional tuning and crystal modification. Subsequently, the microstructure, interphase, and electrolyte should be engineered, and finally, the charging protocol should be optimized. The various LMR chemistries with different Li to *TM*, Ni to Mn, and Co to Ni ratios are techno‐economically analyzed, and perspectives on the ideal LMR composition are presented. Ultimately, the specific energy, energy density, and costs of LMR || graphite cells are compared to state‐of‐the‐art cell chemistries.

## Graphical abstract

Li/Mn‐rich layered oxide cathodes should be designed systematically according to their phase diagram. Starting from a common composition (point 1), the Li‐richness can be reduced (point 2), the Co content can be lowered (point 3), and finally, the Ni to Co ratio can be adjusted. For a more comprehensive evaluation, comparing LMR at an equal delithiation degree is recommended.

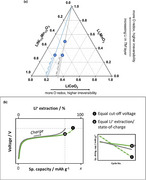

## 1 Introduction

The rising demand for affordable and long‐range electric vehicles necessitates the development of high‐energy lithium ion batteries (LIBs).^[^ ^1^, ^2^, ^3^ ^]^ Among various cathode active materials (CAMs), Li‐rich layered oxides (LRLO), for example the so‐called Li/Mn‐rich layered oxides (*x*Li_2_MnO_3_ · (1−*x*)Li*TM*O_2_; *TM* = Ni, Co, Mn; further referred to as LMR) offer a promising pathway to achieve both, low‐cost and high gravimetric/volumetric energy given the high practical capacity (specific discharge capacities >250 mAh g^−1^) and reasonable mean discharge potential (>3.5 V versus Li|Li*^+^*).^[^ ^4^, ^5^, ^6^ ^]^

Compared to state‐of‐the‐art (SOTA) LiNi*_x_* Co*_y_* Mn*_z_* O_2_ (NCM, *x*+*y*+*z* = 1)‐based cathodes, where capacity originates mainly from transition metals (*TM*s) redox, the Li_2_MnO_3_ component in LMR delivers large capacity from oxygen redox, accessible at high charging potentials (4.6–4.8 V vs Li|Li*^+^*).^[^ ^4^, ^7^, ^8^, ^9^, ^10^ ^]^ Nevertheless, the high oxygen redox potential leads to several (closely intertwined) issues, including oxygen release, voltage hysteresis, redox couple shift, phase transformation, voltage fade, and *TM* dissolution.^[^ ^8^, ^11^, ^12^, ^13^, ^14^, ^15^ ^]^ In practical Li ion cells, oxygen release can rupture pouch cells while *TM* dissolution can damage the anode over the course of electrode crosstalk, where deposited *TM*s enhance local resistance and trigger high surface area lithium (HSAL) plating.^[^ ^16^, ^17^ ^]^ Given the reactivity of HSAL, the amount of lithium inventory depletes throughout cycling, leading to a capacity fade, and in the worst case, to a “sudden death” or “rollover” failure.^[^ ^18^, ^19^ ^]^

The history of LMRs, the charge/discharge mechanism, and the associated issues have been compiled in several review articles.^[^ ^4^, ^7^, ^20^, ^21^, ^22^, ^23^, ^24^, ^25^ ^]^ Furthermore, some articles have comprehensively summarized various modification strategies involving crystal engineering, microstructure modifications, composition tuning, doping, coating, and electrolyte engineering.^[^ ^4^, ^20^, ^22^, ^23^, ^26^, ^27^, ^28^ ^]^ Nevertheless, despite the numerous R&D activities, LMR‐based LIBs are not commercialized, yet.^[^ ^29^ ^]^ Moreover, although Ford and General Motors recently announced their plans to bring LMR‐based cells to the market,^[^ ^30^, ^31^ ^]^ details regarding practical LMR composition and improvement strategies are still limited.

This work first summarizes the mechanistic working principle and failure mechanism of LMR, particularly in comparison with SOTA layered oxide cathodes. Following this discussion, the article provides perspectives on LMR R&D strategy, which should initially focus on compositional tuning and crystal modification, followed by microstructure, surface, and electrolyte engineering, and finally charging protocol optimization. Various LMR compositions with different Li to *TM*, Ni to Mn, and Co to Ni ratios are techno‐economically analyzed, and ideal LMR compositions are discussed, offering the most comprehensive analysis with regard to the vast design opportunities of LMR composition.^[^ ^32^ ^]^ Finally, the specific energy, energy density, and costs of LMR || graphite cells are compared to SOTA LiNi_0.8_Co_0.1_Mn_0.1_O_2_ (NCM811)‐ and LiFePO_4_ (LFP)‐based LIBs.

## 2 Redox Processes of LMR versus NCM

**Figure 1:**
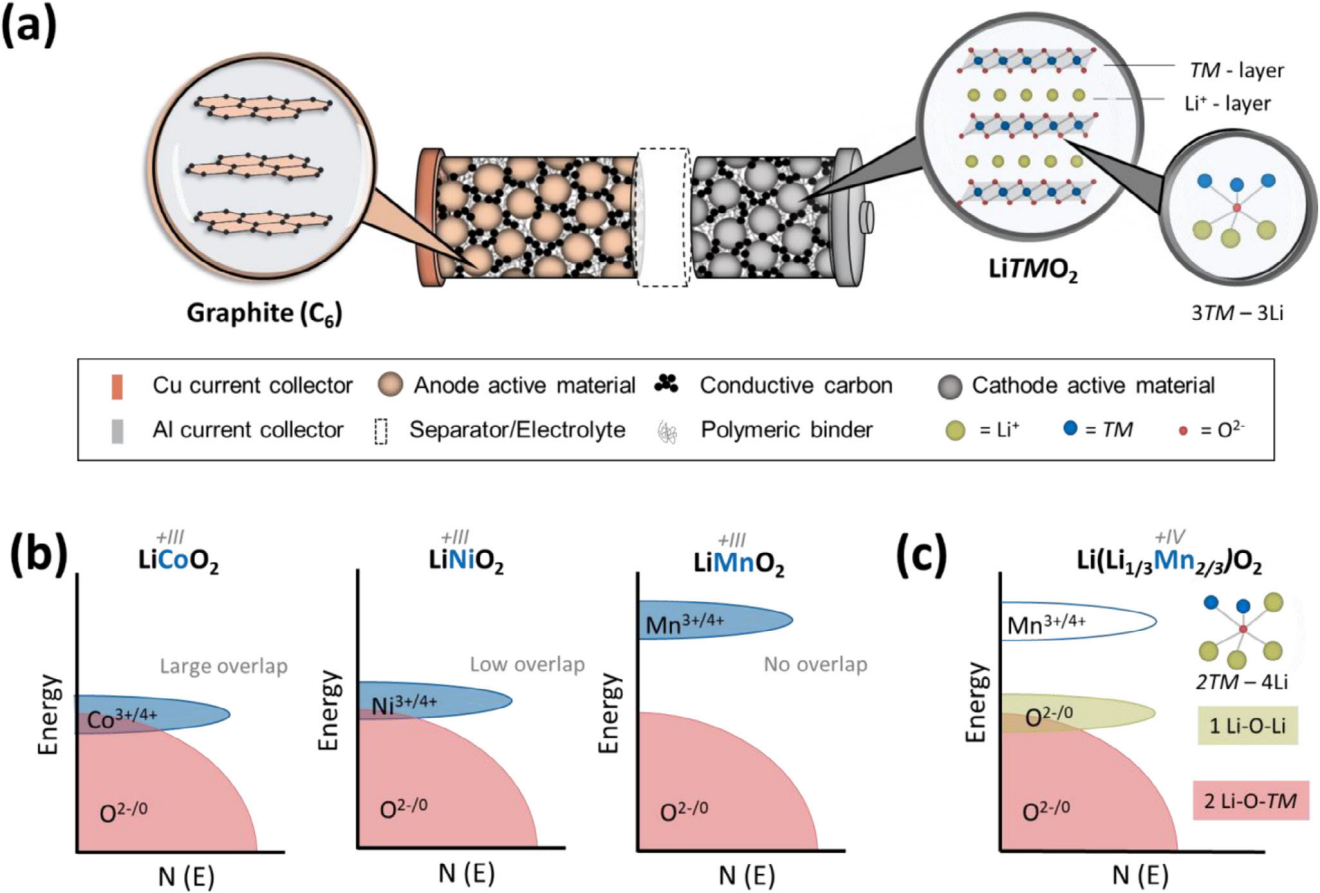
a) In conventional LIBs with graphite‐based anode, the cathode is frequently based on layered oxides, e.g., NCM, where the Li‐ and *TM*‐layer alternate. b) According to the electronic band structure, Co and Ni can lead to irreversible oxygen release after exceeding a certain SOC due to partial band overlap with the lattice oxygen band, while Mn is regarded as chemically stable.^[^ ^34^ ^]^ c) Replacing Mn with Li in the *TM*‐layer up to 1/3 forms Li_2_MnO_3_, where Mn is tetravalent and redox activity entirely stems from lattice oxygen oxidation.

In LIBs with Li*TM*O_2_ (e.g., NCM)‐based cathodes, energy is stored when lithium ions are extracted from NCM, which is balanced by their intercalation into the graphite anode (**Figure** **1**a). In a layered Li*TM*O_2_ the charging process mostly involves electron extraction from the *TM* band and vice versa. Nevertheless, the electron bands of some *TM*s in layered oxides, e.g., LiCoO_2_ or LiNiO_2_, overlap with the oxide band (Figure 1b), allowing for partial participation of oxygen ions in the redox processes,^[^ ^33^ ^]^ which can lead to oxygen evolution and concomitant cathode degradations when a threshold delithiation degree/Li^+^extraction ratio is exceeded.^[^ ^34^ ^]^ Therefore, charging is limited to a threshold state‐of‐charge (SOC),^[^ ^34^ ^]^ which is typically controlled via upper cut‐off voltages (UCV), e.g., < 4.2 V for LiCoO_2_‐based LIBs, corresponding to a Co oxidation state of +3.5 and 50% Li^+^ extraction ratio.^[^ ^34^ ^]^

Although the Mn^3+/4+^ band in LiMnO_2_ has no overlap with the oxide band that can cause chemical instability (Figure 1b),^[^ ^34^ ^]^ charge/discharge process in this material is poorly reversible as the Jahn–Teller active trivalent Mn destabilizes the crystal.^[^ ^35^, ^36^, ^37^ ^]^ Structurally more stable layered Li‐Mn‐O oxide can be formed by replacing 1/3 of the Mn in the *TM*‐layer with Li, to produce Li(Li_1/3_Mn_2/3_)O_2_ (Li_2_MnO_3_) where Mn is tetravalent.^[^ ^38^, ^39^, ^40^ ^]^ Although Mn^4+^ is electrochemically inactive within the practical electrochemical window of LIB cathodes,^[^ ^7^, ^41^, ^42^ ^]^ the Li‐O‐Li configuration from Li occupying the *TM* layer forms a distinct non‐bonding oxygen band located at higher energy levels than that of the Li‐O‐*TM* band (Figure 1c).^[^ ^7^, ^43^ ^]^ This allows for lattice oxygen oxidation in Li_2_MnO_3_ to proceed at lower voltages and with lower risks of structural destabilization than in stoichiometric layered oxide.^[^ ^7^, ^43^ ^]^ Given the lower molar mass of Li_2_MnO_3_ (77.9 g mol^−1^) versus NCM 811 (97.3 g mol^−1^), Li_2_MnO_3_ can achieve a higher theoretical specific charge capacity of 344.1 mAh g^−1^ versus 275.4 mAh g^−1^ for NCM 811. When additional delithiation from the *TM* layer is considered, even a specific capacity of 457.6 mAh g^−1^ can theoretically be obtained.

**Figure 2:**
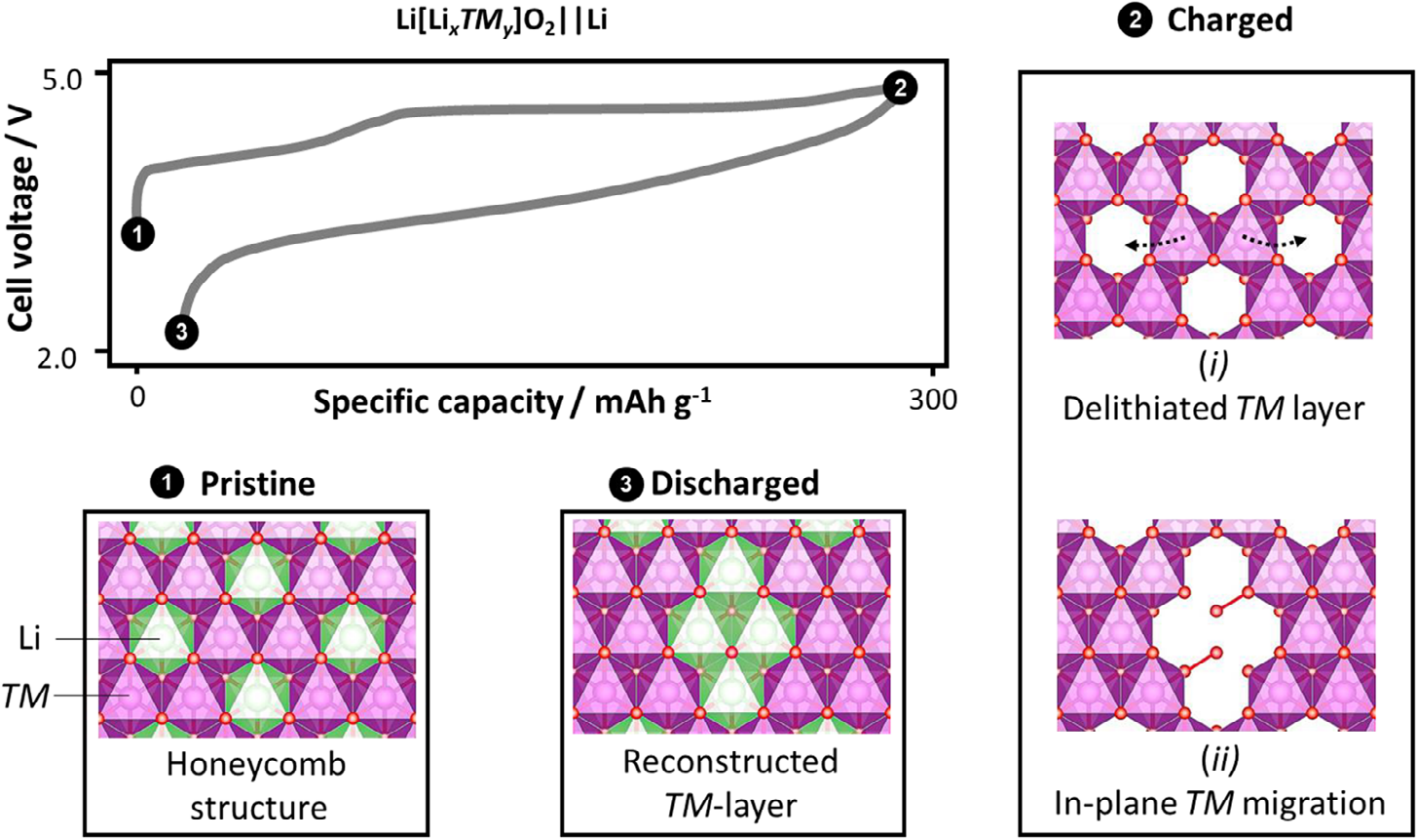
A typical 1st cycle voltage versus capacity profile of LMR || Li and the corresponding crystallographic arrangement of the *TM* layer. The ordering of Li/Ni and Co/Mn in the *TM* layer gives rise to the honeycomb superstructure ordering in pristine LMR. Lattice oxygen oxidation is accompanied by the delithation of the *TM* layer, which triggers in‐plane *TM* migration to Li^+^ vacancies, resulting in vacancy clusters and molecular O_2_ formation. Upon discharge, molecular O_2_ is reduced and Li^+^ return to the *TM* layer, but in different sites compared to the pristine structure. This new coordination structure has higher energy compared to the honeycomb‐ordered pristine state, resulting in a lower charge and discharge voltage.^[^ ^11^, ^46^ ^]^

**Figure 3:**
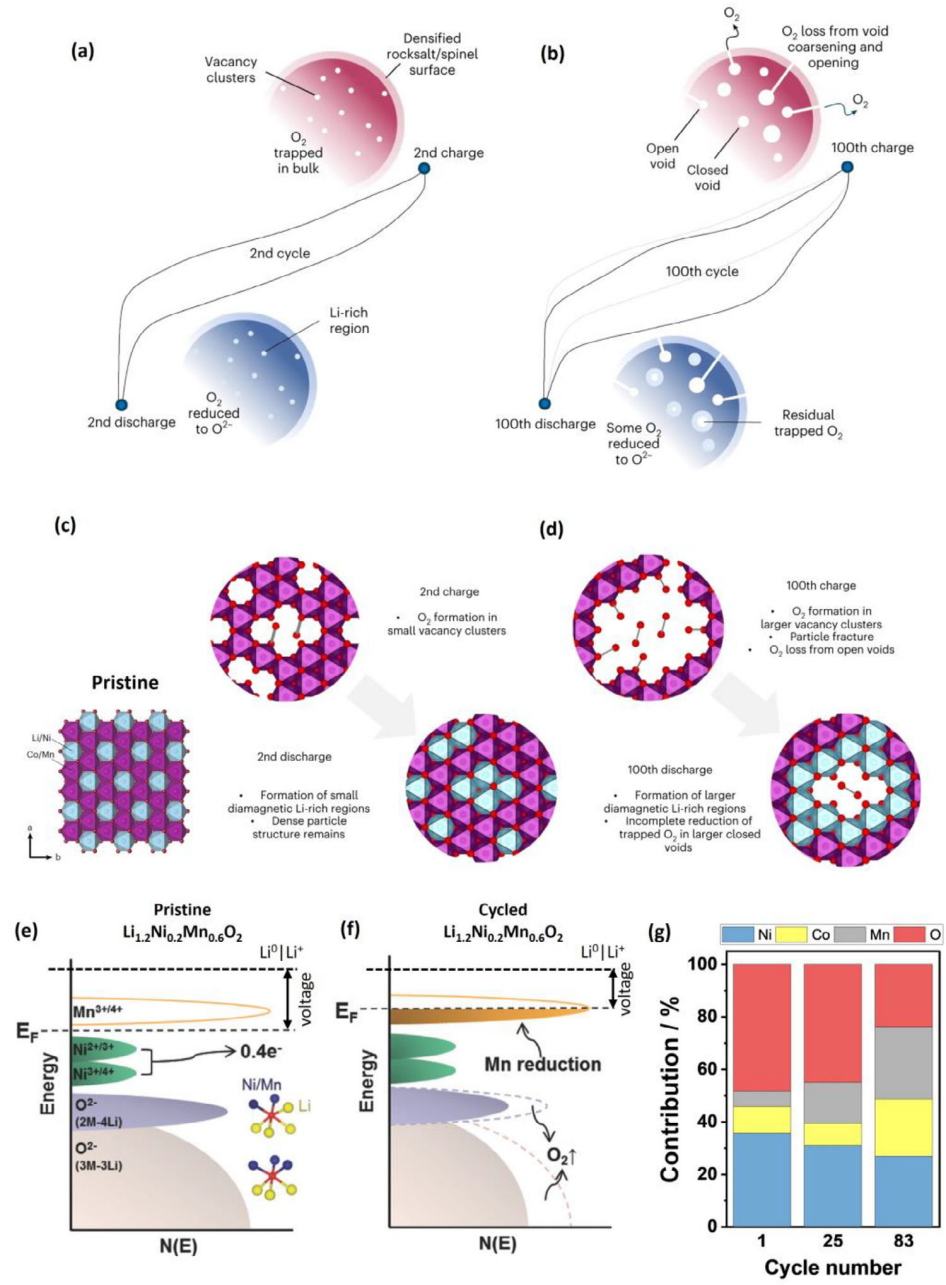
Macroscopic changes in an LMR primary particle in the a) 2nd and b) 100th cycle, and the corresponding voltage profiles. Changes in the LMR *TM* layer structure at fully charged and discharged states, associated with O_2_ and vacancy cluster formation in the c) 2nd and d) 100th cycle. e) LMR band structure having two types of oxygen bands from the 2M‐4Li and 3M‐3Li configuration. The 2M‐4Li structure arises from the presence of Li in the TM layer, enabling electron extraction from this oxygen band. f) Cycled LMR band structure showing partial loss of oxygen that is compensated by Mn reduction, activating Mn^3+|4+^ redox that operates at lower potentials. g) Redox couple contribution of Ni, Co, Mn, and O in LMR at different cycle numbers.^[^ ^12^ ^]^ a–d) Reproduced with permission.^[^ ^46^ ^]^ Copyright 2024, Springer Nature. e,f) Reproduced with permission.^[^ ^47^] Copyright 2017, Wiley‐VCH GmbH.

Considering that electrochemical activity in Li_2_MnO_3_ is poorly reversible, R&D rather focuses on the composite of Li_2_MnO_3_ and Li*TM*O_2_, literature‐known and termed as LMR throughout this manuscript.^[^ ^39^, ^44^ ^]^ Nevertheless, LMR issues remain, such as first‐cycle voltage hysteresis, long‐term voltage fade associated with irreversible oxygen loss, redox couple shift, phase transformation, as well as *TM* dissolution,^[^ ^11^, ^12^, ^13^, ^15^, ^45^ ^]^ hindering its practical implementation in high‐energy LIBs so far. The large voltage hysteresis of LMR in the first cycle is assigned to pronounced structural rearrangement upon oxide oxidation by Bruce et al.,^[^ ^11^, ^12^ ^]^ which remains afterward (**Figure** **2**).^[^ ^11^ ^]^ Over the course of in‐plane *TM* migration, the oxidized lattice oxygen can form strong sigma interactions with adjacent oxygen ions, producing molecular O_2_ (Figure 2).^[^ ^11^, ^46^ ^]^ On the surface, molecular O_2_ is irreversibly released, forming a densified rock‐salt/spinel surface layer, while in the bulk, they are confined in vacancy clusters (**Figure** **3**a)^[^ ^11^, ^46^ ^]^ and can be reversibly reduced back to O^2−^ during discharge.^[^ ^11^, ^46^ ^]^

The severe voltage hysteresis over the course of in‐plane TM rearrangement predominantly occurs in the first cycle (Figure 3a,c). However, after several cycles, O_2_ can evolve due to particle cracking and void opening. In addition, this void coarsening can further increase Li‐rich regions (light blue octahedra) and suppress O_2_ reduction (Figure 3b,d). The more oxygen disappears, the higher the voltage fade due to increasing Mn^3+/4+^ redox.^[^ ^46^ ^]^

Electrochemically, irreversible oxygen release is charge‐compensated by Mn^4+^ reduction, activating the Mn^4+^/Mn^3+^ redox couple that is located at a higher Fermi level than the Ni redox couple (Figure 3e,f). This decreases the operating voltage of LMR, known as voltage fade.^[^ ^12^, ^47^ ^]^ According to Amine et al. the capacity contribution of Mn^3+/4+^ and Co^2+/3+^ redox couples (Figure 3g) gradually increases over the course of irreversible oxygen release throughout cycling.^[^ ^12^, ^48^ ^]^ Additionally, out‐of‐plane *TM* migration can cause the layered‐to‐spinel phase transformation and also contribute to voltage fade.^[^ ^49^, ^50^ ^]^ According to Kleiner et al., *TM* migration to the tetrahedral Li sites is reversible, but irreversible when migrating to the octahedral Li sites.^[^ ^49^, ^50^ ^]^

## 3 Composition Tuning

The reversibility of oxygen redox in LMR depends on LMR composition (**Table** **1**), necessitating the optimization of Li to *TM*, Ni to Mn, and Co to Ni ratios.^[^ ^6^, ^47^, ^51^, ^52^, ^53^ ^]^ Here, the different LMR chemistries are techno‐economically analyzed using the Battery Performance and Cost Model (BatPaC) to identify the best compromise in terms of costs and performance (see Note S1, Supporting Information).^[^ ^54^, ^55^ ^]^ The cell‐level energy density, specific energy, and cost are calculated based on the default cell design parameters for NCM 811 || graphite (energy) cells with some adjustments listed in Tables S1 and S2 (Supporting Information). The calculation employs electrochemical data from previous reports and approximated LMR powder price (Tables S3–S7, Supporting Information).^[^ ^6^, ^15^, ^47^, ^51^, ^52^, ^53^ ^]^ It must be underlined that the energy and cost values presented here are based on the first cycle discharge capacity, without any consideration of cycle life.

**Table 1:** Summary of LMR electrochemical performance with varied chemical compositions and crystal structures. The number (#) sign indicates that the values are obtained at a specific current of 20 mA g^−1^. The asterisk (*) sign indicates reported values in the reference articles. Otherwise, they are extracted from the respective figures. The dash (‐) symbol indicates that no data is reported. The Li extraction ratio is calculated based on the charge capacity and theoretical delithiation capacity of Li in the Li layer.

Modification		Example compound	1st dischg. cap. (4.8 V)	1st cyc. CE / %	Capacity retention / %	Initial dischg. voltage / V	Avg. dischg. voltage retention	Condition	*TM* migration	Oxygen release	Li^+^ extraction / %	Reference
Li:TM ratio	Li‐poor	Li_1.14_ *TM*_0.86_O_2_	268*	85*	97 (47 cyc)	3.60 (Avg)	98.2% (47 cyc)	25 °C 0.2 C	low	0	104.6	[52]
	Intermediate	Li_1.17_ *TM*_0.83_O_2_	287*	86*	93 (47 cyc)	3.52 (Avg)	98.4% (47 cyc)		medium	30 µmol m^−2^_AM_	107.7	
	Li‐rich	Li_1.20_ *TM*_0.80_O_2_	283*	83*	88 (47 cyc)	3.48 (Avg)	99.1% (47 cyc)		high	85 µmol m^−2^_AM_	109.0	
Ni:Co ratio	Ni‐rich	Li_1.2_Ni_0.2_Mn_0.6_O_2_	240	78	92 (50 cyc)	3.6 (50% SoC)	High (50 cyc)	RT 20 mA g^−1^	low	low	97.6	[51]
	Intermediate	Li_1.2_Ni_0.13_Co_0.13_Mn_0.54_O_2_	284	82	82 (50 cyc)	3.5 (50% SoC)	Medium (50 cyc)		–	–	110.2	
	Co‐rich	Li_1.2_Co_0.4_Mn_0.4_O_2_	226	67	75 (50 cyc)	3.5 (50% SoC)	Low (50 cyc)		high	high	108.1	
Ni:Co ratio	Co/Ni = 0	Li_1.13_Ni_0.275_Mn_0.580_O_2_	268	88*	91 (100 cyc)	3.71 (Avg)	97% (100 cyc)	30 °C 0.5 C (100 mA g^−1^)	low	very low	99.8	[6]
	Co/Ni = 0.5	Li_1.13_Ni_0.181_Co_0.089_Mn_0.560_O_2_	282	86*	93 (100 cyc)	3.63 (Avg)	91% (100 cyc)		medium	very low	105.8	
	Co/Ni = 1	Li_1.13_Ni_0.130_Co_0.135_Mn_0.551_O_2_	286	90*	90 (100 cyc)	3.51 (Avg)	89% (100 cyc)		high	medium	101.6	
	Co/Ni = 2	Li_1.13_Ni_0.086_Co_0.174_Mn_0.544_O_2_	279	90*	73 (100 cyc)	3.48 (Avg)	88% (100 cyc)		high	high	98.4	
Ni:Mn ratio	Mn‐rich	Li_1.2_Ni_0.2_Mn_0.6_O_2_	270^#^	75	87 (50 cyc)	3.71 (Avg)	98% (50 cyc)	60 °C 1 C	high	–	113.7	[47]
	Intermediate	Li_1.2_Ni_0.3_Mn_0.5_O_2_	253^#^	72	92 (50 cyc)	3.77 (Avg)	98.2% (50 cyc)		–	–	112.2	
	Ni‐rich	Li_1.2_Ni_0.4_Mn_0.4_O_2_	242^#^	71	90 (50 cyc)	3.88 (Avg)	98.9% (50 cyc)		low	–	109.8	
Layer stacking	O3 (Gen 1)	Li(Li_0.2_Ni_0.2_Mn_0.6_)O_2_	217	75	75 (40 cyc)	3.53 (Avg)	96.6% (40 cyc)	RT 5 mA g^−1^	irreversible	–	92.5	[15]
	O2 (Gen 2)	Li_0.83_(Li_0.2_Ni_0.2_Mn_0.6_)O_2_	222	94	78 (40 cyc)	3.53 (Avg)	98.6% (40 cyc)		reversible	–	91.6	

The extent of Li_2_MnO_3_, i.e., “Li‐richness” or Li to *TM* ratio, influences oxygen redox behavior. For example,^[^ ^52^ ^]^ the comparison of 0.33 Li_2_MnO_3_ · 0.67 LiMeO_2_ (Li_1.14_ *TM*_0.86_O_2_), 0.42 Li_2_MnO_3_ · 0.58 LiMeO_2_, (Li_1.17_ *TM*_0.83_O_2_) and 0.50 Li_2_MnO_3_ · 0.50 LiMeO_2_ (Li_1.20_ *TM*_0.80_O_2_), shows that the commonly investigated LMR composition (Li_1.20_ *TM*_0.80_O_2_) apparently performs worst (Table 1; LiMeO_2_ likely LiNi_0.38_Co_0.21_Mn_0.41_O_2_).^[^ ^4^, ^52^ ^]^ Although Li_1.20_ *TM*_0.80_O_2_ delivers the highest charge capacity due to more extensive oxygen oxidation, its low CE results in a first‐cycle discharge capacity that is lower than Li_1.17_ *TM*_0.83_O_2_.^[^ ^52^ ^]^ Moreover, an excessive oxygen release, low capacity retention, and large voltage are observed.^[^ ^52^ ^]^ Worth noting, the low reversibility of Li_1.20_ *TM*_0.80_O_2_ may stem from its higher degree of delithiation (Li^+^ extraction ratio) compared to the other compositions when charged to the same UCVs (Table 1), which should be reassessed by charging to similar Li^+^ extraction ratios, as done previously for Ni‐rich versus Ni‐poor and polycrystalline versus single crystalline NCMs.^[^ ^56^, ^57^ ^]^

**Figure 4:**
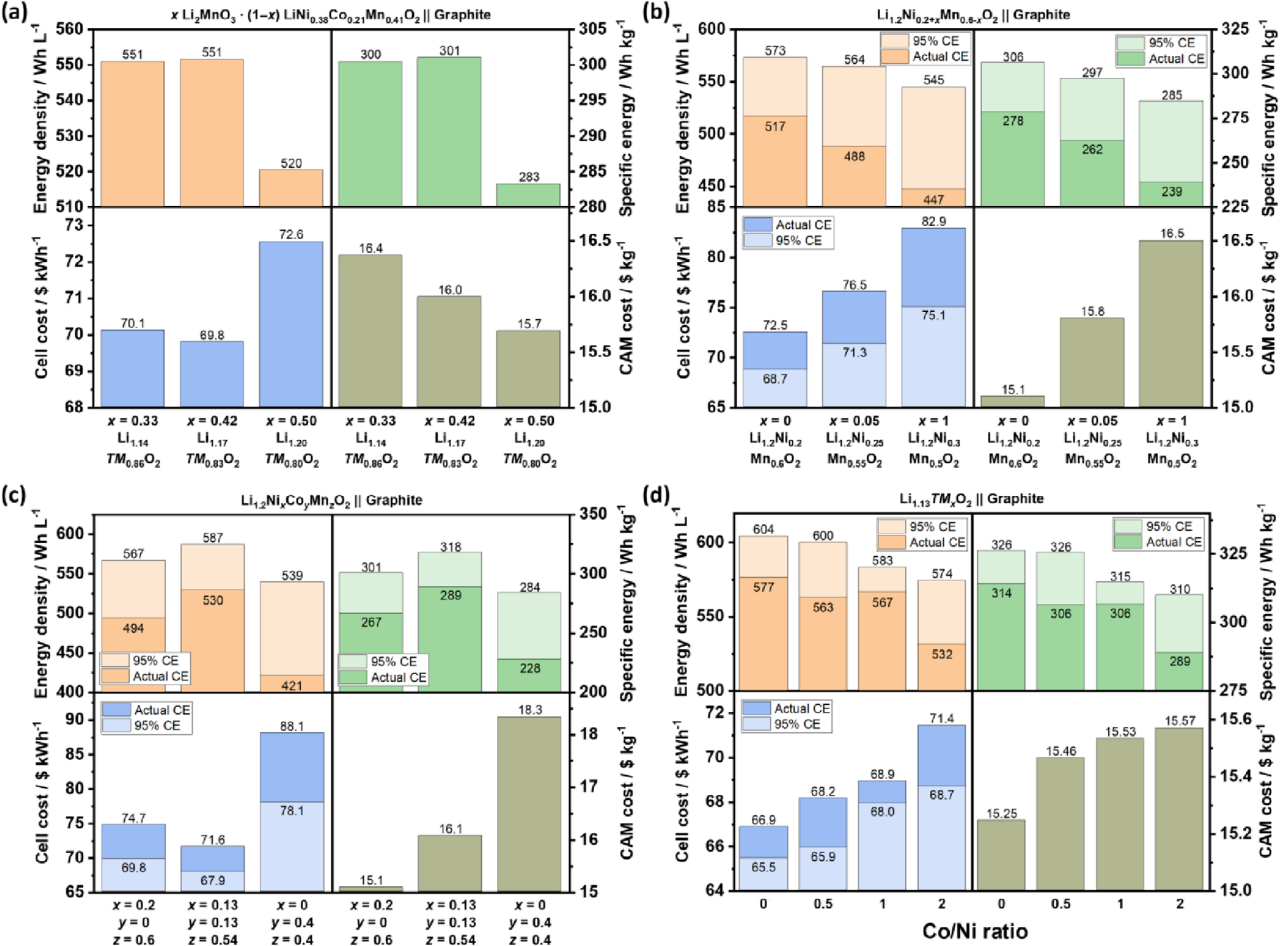
Techno‐economic analysis of LMR || graphite cells with varied a) Li_2_MnO_3_ content/Li‐richness, b) Ni to Mn ratio/Ni oxidation state, c) Co/Ni ratio for Li_1.2_ *TM*_0.8_O_2_, d) Co/Ni ratio for Li_1.13_ *TM_x_* O_2_. This figure presents energy density and specific energy values at the cell level. Considering the low Coulombic efficiencies (CEs) of LMR chemistries presented in (b–d) due to the unoptimized and impractical sol–gel synthesis,^[^ ^58^ ^]^ additional energy and cost values assuming a high CE of 95% are presented in (b–d). It is notable that when the actual CE values (lower) are used, the energy density and specific energy values are lower, while the cell costs are higher, as more graphite is needed to accommodate the large charge capacities in the formation cycle.

Due to the lower discharge capacity, the Li_1.20_ *TM*_0.80_O_2_ || graphite cell delivers lower energy density/specific energy and a higher cell cost than cells with Li_1.14_ *TM*_0.86_O_2_ and Li_1.17_ *TM*_0.83_O_2_ (**Figure** **4**a). Although employing Li_1.17_ *TM*_0.83_O_2_ results in a slightly lower cost than Li_1.14_ *TM*_0.86_O_2_, which stems from the lower CAM cost due to the lower cost per mol of lithium versus nickel and cobalt (Table S7, Supporting Information), Li_1.17_ *TM*_0.83_O_2_ experiences more intense O_2_ release in the initial cycles (Table 1), making it impractical for commercial use. Nevertheless, despite the lower amount of gas produced by Li_1.14_ *TM*_0.86_O_2_ than Li_1.17_ *TM*_0.83_O_2_, the cycle life of pouch cells employing the former composition charged to a UCV of 4.6 V is still limited by cell rupture after 250 cycles due to severe gassing.^[^ ^16^, ^52^ ^]^

Variation of Ni to Mn ratio in Co‐free LMR alters the Ni oxidation states and the oxygen redox behavior.^[^ ^47^, ^53^, ^59^ ^]^ An increase of Ni oxidation state from 2+ in Li_1.2_Mn_0.6_Ni_0.2_O_2_ (Li_2_Mn^4+^O_3_·LiNi^2+^_0.5_Mn^4+^_0.5_O_2_) to 3+ in Li_1.2_Mn_0.4_Ni_0.4_O_2_ (Li_2_Mn^4+^O_3_·LiNi^3+^O_2_) allows for the extension of Ni redox activity from Ni^3+^|Ni^4+^ to Ni^2+^|Ni^4+^ after oxygen release in the first charge.^[^ ^47^ ^]^ In other words, Ni^3+^ acts as a buffer before Mn^3+^|Mn^4+^ redox is activated, which enhances capacity retention,^[^ ^53^, ^59^ ^]^ and increases LMR discharge voltage and its retention (Table 1; Table S4, Supporting Information).^[^ ^47^, ^53^ ^]^

Nevertheless, the energy density and specific energy decrease with increasing Ni content (Figure 4b), due to lower discharge capacity (Table S2, Supporting Information), also in Co‐containing LMR (Figure S1a, Supporting Information).^[^ ^53^ ^]^ At the same time the substitution of cheap Mn with expensive Ni increases CAM and cell cost (Figure 4 b). Despite this, increased Ni content can be beneficial for operations at 60 °C, where first cycle capacity reduction is less pronounced and the suppression of voltage fade is even more pronounced than at ambient temperature (Figure S1b and Tables S2,S4, Supporting Information).^[^ ^47^ ^]^

**Figure 5:**
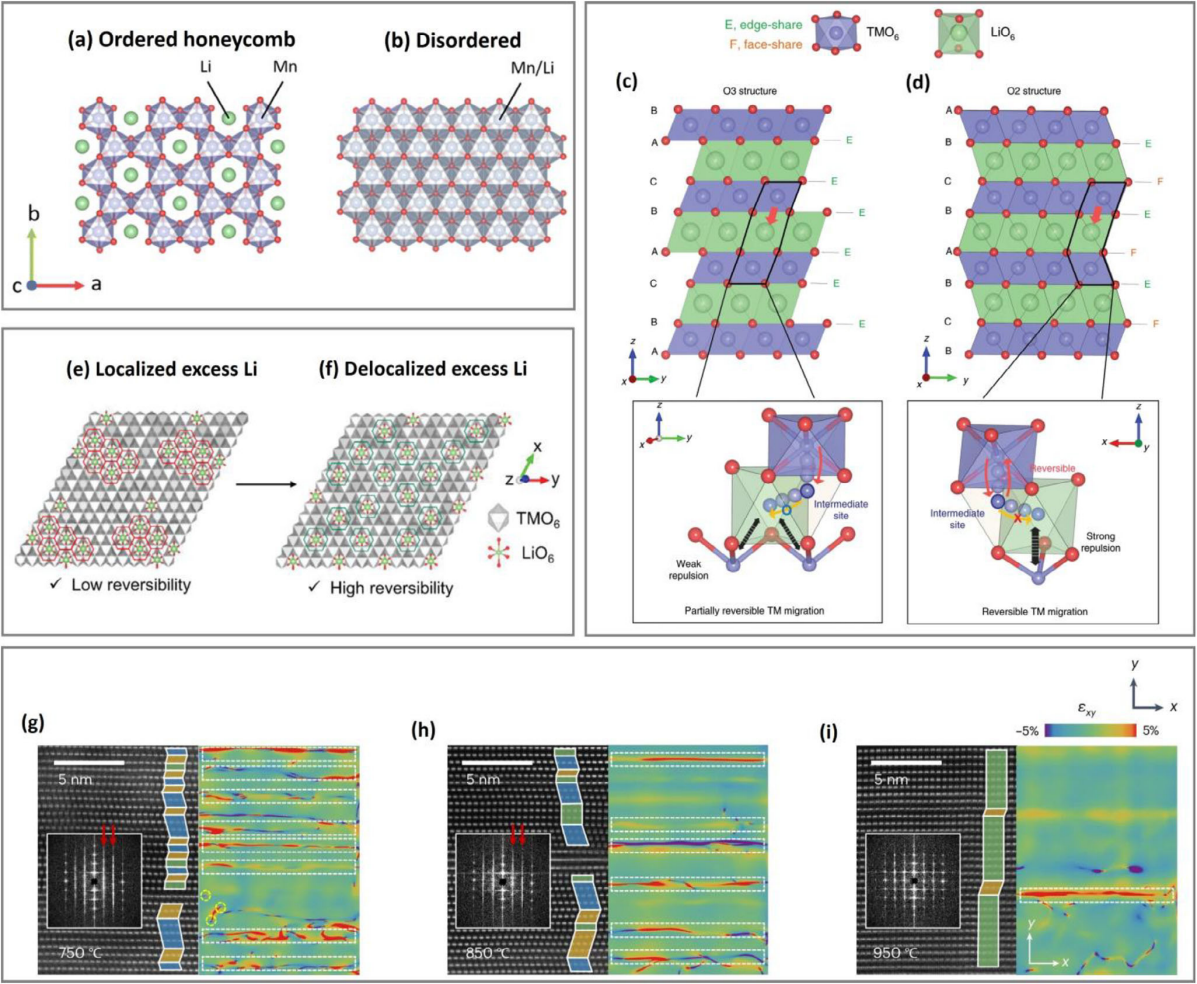
a) Honeycomb‐ordered and b) disordered *TM* layer in LMR.^[^ ^14^ ^]^ Layer stacking in c) O3 structure with irreversible *TM* migration and d) O2 structure with reversible *TM* migration.^[^ ^15^ ^]^ e) Localized and f) delocalized excess Li (Li_2_MnO_3_) in the *TM* layer of LMR.^[^ ^63^ ^]^ STEM/GPA stacking sequence analysis results of pristine O2‐type LMR synthesized at g) 750, h) 850, and i) 950 °C. The insets show the fast Fourier transform (FFT) patterns of the respective STEM images, and the red arrows point at streak lines indicating disordered *TM* layers, which are absent in (i). The blue and yellow tapezoid indicates layer stacking with RSF, and the green trapezoid indicates RSF‐free stacking. The white dotted boxes in the GPA maps indicate areas with high concentration of mechanical strain (*ε*_xy_).^[^ ^78^ ^]^ a,b) Reproduced with permission.^[^ ^14^ ^]^ Copyright 2023, Royal Society of Chemistry. c,d,g–i) Reproduced with permission.^[^ ^15^, ^78^ ^]^ Copyright 2020, 2024, Springer Nature. e,f) Reproduced with permission.^[^ ^63^] Copyright 2021, Wiley VCH GmbH.

Adjusting the Co content alters the ordering in the *TM* layer, as illustrated in **Figure** **5**a,b. The O2‐type Li*_x_* [Li_0.25_Mn_0.625_Ni_0.125_]O_2_ and Li*_x_* [Li_0.25_Mn_0.5_Co_0.25_]O_2_ LMR forms *TM* layers with honeycomb and disordered structure, respectively.^[^ ^14^ ^]^ In the latter case, Co^3+^ prohibits superstructure ordering in the *TM* layer, allowing for more *TM* migration pathways that lead to more severe voltage decay than the former, despite the structurally stable O2‐type stacking. In contrast, Ni^2+^ substitution forms an ordered honeycomb structure, capable of hindering *TM* migration, thereby stabilizing oxygen redox. The suppression of *TM* migration, particularly in the Li layer, facilitates better Li diffusion kinetics during lithiation, allowing for more facile discharge kinetics in Ni‐based compared to Co‐based O2‐type LMR.^[^ ^14^, ^60^ ^]^

A similar improvement in structural stability enabled by Ni substitution is also observed in O3‐type Li_1.2_Mn_0.6_Ni_0.2_O_2_ versus Li_1.2_Mn_0.4_Co_0.4_O_2._^[^ ^51^ ^]^ Nevertheless, Co^4+^ formed during charging can better facilitate oxygen redox than Ni^3+^/Ni^4+^, likely because of in‐plane *TM* movement leading to more voids for oxygen (see Figure 2), thus enabling higher charge capacities for Co‐based LMR, though the low CE of this material lowers discharge capacity (Table 1).^[^ ^51^ ^]^ Given that *TM*s migrate to the tetrahedral Li sites during charge, it is possible that Ni^4+^ migrates less due to its higher stability in octahedral sites, as it has lower values of crystal field stabilization energy (CFSE) because of d6 electron configuration, meaning a fully occupation of the three *t_2g_* orbitals compared to Co^4+^ (d5 electrons only). Nevertheless, due to the presence of Li vacancy in the *TM* layer and oxidized lattice oxygen, further validation of CFSE theory in LMR is needed.

The contrasting role of Co and Ni underlines the importance of compositional tuning to balance oxygen redox and stability. For O3‐type LMR, Co‐containing Li_1.2_Ni_0.13_Co_0.13_Mn_0.54_O_2_ delivers higher capacity and first cycle CE than Co‐free Li_1.2_Ni_0.2_Mn_0.6_O_2_, with a compromise in capacity and voltage retention.^[^ ^6^, ^51^ ^]^ As a result, the LMR cell with Li_1.2_Ni_0.13_Co_0.13_Mn_0.54_O_2_ delivers higher energy density and specific energy at a lower cell cost than Li_1.2_Ni_0.2_Mn_0.6_O_2_ and Li_1.2_Co_0.4_Mn_0.4_O_2_ (Figure 4c).

For LMR with a lower Li content, i.e., Li_1.13_ *TM_x_* O_2_, an increase in Co/Ni ratio appears not to increase energy density and specific energy (Figure 4d), as the initial discharge capacity of LMR with Co/Ni = 1 is only 18 mAh g^−1^ higher than that with Co/Ni = 0,^[^ ^6^ ^]^ whereas in Li_1.2_ *TM_x_* O_2_, the discharge capacity is 44 mAh g^−1^ higher for Co/Ni = 1 (Table 1).^[^ ^51^ ^]^ It must also be noted that in the former case,^[^ ^6^ ^]^ the Ni remains in a 2+ state due to some lithium vacancies in the evaluated compositions (Table S8, Supporting Information), while in the latter case,^[^ ^51^ ^]^ Ni stays in a 2+ state due to Mn content adjustment.

Although Figure 4d suggests that Co‐free LMR with a composition of Li_1.13_Ni_0.275_Mn_0.580_O_2_ is the most interesting O3‐type LMR chemistry due to its high energy, low cost, and low gassing,^[^ ^6^ ^]^ further validation of fully lithiated LMR with a constant Ni oxidation state of 2+ is necessary. In this case, employing a low Co/Ni ratio would increase the Ni oxidation state and consequently alter the redox buffering behavior (Table S9, Supporting Information).^[^ ^47^, ^53^ ^]^ Given the detrimental effect of redox buffering on first‐cycle CE and discharge capacity, a Co‐free LMR may eventually deliver lower energy than those with higher Co/Ni ratios. Considering that *TM* composition affects reversible capacity, i.e., delithiation degree (Table 1), a fair comparison of LMRs with different *TM* compositions charged to the same delithiation degree is also required to choose the most ideal LMR chemistry.^[^ ^56^, ^57^ ^]^ Furthermore, given the strong influence of first cycle CE on N/P ratio, and consequently on energy density and cost, future work should focus on comparing different LMR compositions synthesized via an optimized co‐precipitation + calcination method to provide a more reliable charge and discharge capacity input value for the cost calculations.

## 4 Crystal Engineering

Further oxygen redox stability enhancement can be achieved by modifying *TM* migration pathways via crystal engineering,^[^ ^15^, ^61^, ^62^, ^63^, ^64^ ^]^ e.g., by doping,^[^ ^65^, ^66^, ^67^, ^68^, ^69^, ^70^ ^]^ Li delocalization in the *TM* layer,^[^ ^63^, ^64^ ^]^ and layer stacking modification.^[^ ^15^, ^62^, ^71^ ^]^ Doping can alter electronic and crystal structure, leading to improved electrochemical performance.^[^ ^68^, ^70^, ^72^ ^]^ The impact of different dopants on LMR performance has been comprehensively summarized by Yang *et.al*.^[^ ^4^ ^]^ Another approach to stabilizing oxygen redox centers involves delocalizing the excess Li in the *TM* layer (Figure 5e,f), as localization of Li sites in the *TM* layer triggers detrimental interaction between oxidized oxygen ions, resulting in pronounced irreversibility.^[^ ^63^, ^64^ ^]^

Delocalization of Li_2_MnO_3_ domains can be achieved via increasing the uniformity of cation distribution of precursor during synthesis, e.g., via sol–gel method, instead of solid‐state.^[^ ^73^ ^]^ Also, calcination with LiOH·H_2_O instead of Li_2_CO_3_ is reported to increase delocalization.^[^ ^74^ ^]^ Nonetheless, the impact of doping and Li delocalization on oxygen redox is rather limited as the former only affects the local structure of the crystal,^[^ ^65^, ^68^ ^]^ and the latter does not fundamentally address the inevitable *TM* migration issue.^[^ ^63^ ^]^ Hence, rather than local modificattions, modifying the entire crystal should be more effective (**Table** **2**).

**Table 2:** Types of LMR, their properties, and their synthesis method.

LMR type	Crystal structure	*TM* layer ordering	*TM* in Li layer	*TM*	Synthesis	Refs.
Gen 1	O3	ordered/disordered	synthesis defect	Mn, Ni, Co	carbonate co‐precipitation + calcination	[4]
Gen 2	O2	ordered/disordered	synthesis defect	Mn, Ni, Co	carbonate co‐precipitation + calcination + ion‐exchange	[14, 15, 71, 75]
Gen 3	O2	ordered	intentional placement	Mn, Ni	carbonate co‐precipitation with *TM* overdose + calcination + ion‐exchange	[62]

The conventional carbonate‐based co‐precipitation + calcination synthesis method yields LMR with O3‐type layered structure (Gen 1 LMR) as the most thermodynamically preferred structure.^[^ ^71^ ^]^ O3‐type LMR suffers from irreversible *TM* migration due to the weak repulsion experienced by *TM* in the Li layer, stemming from the three repeating layer units in the layered structure (ABC; Figure 5c).^[^ ^15^, ^71^ ^]^ In contrast, a synthesis process involving an intermediate P2‐type Na layered oxides, followed by ion exchange enables the formation of LMR with an O2‐type structure (Gen 2 LMR; Figure 5f).^[^ ^15^, ^71^, ^75^, ^76^ ^]^ O2‐type LMR with two repeating layer units (ABAC) is claimed to have a stronger repulsion towards *TM* occupying the octahedral sites in the Li layer, thus suppressing irreversible *TM* migration.^[^ ^15^ ^]^ As a result, reversible oxygen reduction at high potential is maintained, thereby suppressing first‐cycle voltage hysteresis and long‐term voltage decay.^[^ ^15^, ^76^ ^]^

Further enhancement of the O2‐type LMR is possible via overdosing *TM* carbonates during the carbonate co‐precipitation process, which positions *TM*, likely Ni^2+^, in the Li layer (*TM*_Li_), yielding LMR with a capped honeycomb structure (Gen 3 LMR).^[^ ^62^ ^]^ *TM*_Li_ is argued to pin the unstable O^2−^‐Li^+^ bonds in the honeycomb structure. Despite the absence of *TM*_Li_ according to a thorough structural evaluations in another study,^[^ ^77^ ^]^ this material has a more stable oxygen redox and reduced oxygen release, which is also followed by the maintenance of the honeycomb structure and negligible voltage fade after 50 cycles.^[^ ^62^ ^]^

Finally, the crystal structure can be stabilized by lowering synthesis‐derived defects such as rotational stacking faults (RSFs), i.e., misalignment of the different *TM* layers due to the rotation of the *TM* slab, which induces mechanical strain accummulation between the misaligned layers Figure 5g.^[^ ^78^, ^79^ ^]^ Recently, RSF is shown to promote *TM* slab gliding, oxygen dimerization, and *TM* migration in the nanoscale level and initiate intragranular crack in the microscale level, both exacerbating capacity and voltage fade.^[^ ^78^ ^]^ This electrochemomechanical failure can be suppressed by lowering the amount of RSF via optimizing the synthesis process, *e.g*, via adjusting the calcination temperature, as shown by the scanning transmission electron microscopy (STEM)/geometric phase analysis (GPA) results in Figure 5g–i.^[^ ^78^ ^]^

As summarized in Table 1, Gen 2 LMR appears promising as it exhibits a high first‐cycle Coulombic efficiency (CE) of 94% (vs 75% for Gen 1 LMR).^[^ ^80^, ^81^, ^82^ ^]^ Nevertheless, this high CE needs to be evaluated carefully as O2‐type LMRs are generally lithium‐deficient, i.e.,, some of them deliver low delithiation capacity and can accommodate extra lithium during lithiation.^[^ ^83^, ^84^ ^]^ Given the strong influence of finite lithium inventory on the energy density and cost of practical Li ion cells, further research efforts on reducing the lithium deficiency, developing simpler synthesis methods, exploring pre‐lithiation strategies, and lithium accounting are needed to enable high‐energy, low‐cost, and long‐life LMR || graphite cells.

## 5 Microstructure, Surface, and Electrolyte Engineering

**Figure 6:**
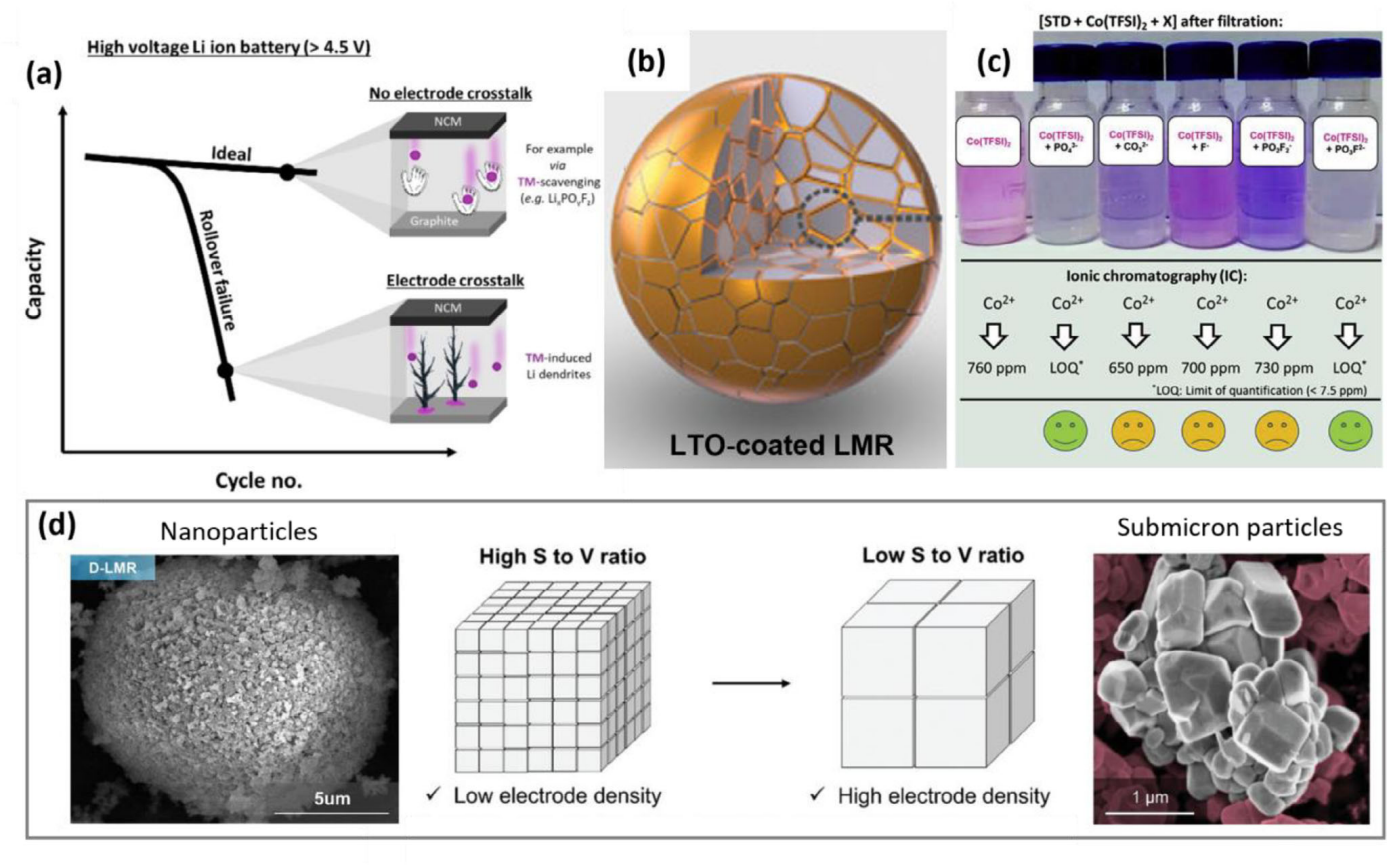
a) Illustration of rollover failure and its prevention via suppressing *TM* deposition.^[^ ^89^ ^]^ b) Infusive coating for full protection of the positive electrode particle surface.^[^ ^45^ ^]^ c) Co^2+^ scavenging effect of PO_4_^3‐^, CO_3_^2−^, F^‐^, PO_2_F^2−^, and PO_3_F^2−^ anions.^[^ ^112^ ^]^ d) Nanoscale and submicron primary particles result in high and low surface‐to‐volume ratios, respectively.^[^ ^63^, ^64^ ^]^ a–d) Reproduced with permission.^[^ ^45^, ^63^, ^64^, ^89^, ^112^] Copyright 2020, 2021, 2022, Wiley VCH GmbH.

The cycle life of high‐voltage LIBs is relevantly limited by electrode crosstalk, which can even lead to rollover failure (**Figure** **6**a).^[^ ^18^, ^85^ ^]^ In this case, the resulting Li inventory loss due to HSAL formation can contribute more to capacity fade than intrinsic cathode degradations.^[^ ^17^, ^86^ ^]^ Therefore, inhibiting *TM* dissolution^[^ ^45^, ^87^, ^88^ ^]^ and/or scavenging dissolved *TM* can be reasonable strategies,^[^ ^89^, ^90^, ^91^ ^]^ e.g., via modifying the cathode particles^[^ ^4^, ^88^, ^92^ ^]^ or the electrolytes.^[^ ^4^, ^87^, ^93^ ^]^ Cathode particles modification includes microstructure engineering (Figure 6d) and/or surface modification (Figure 6b). In LMR, besides the high charging potentials, the large specific surface area also promotes *TM* dissolution.^[^ ^16^, ^17^ ^]^ An example strategy to lower the surface area includes synthesizing submicron particles with low surface‐to‐volume ratios (Figure 6d),^[^ ^63^ ^]^ which can also be deagglomerated to form single crystalline LMR particles.^[^ ^92^, ^94^, ^95^, ^96^, ^97^ ^]^ Interestingly, employing the SOTA hydroxide co‐precipitation method does not form LMR secondary particles that are as dense as NCM.^[^ ^63^, ^96^, ^98^, ^99^ ^]^ Instead, porous LMR secondary particles with a similar specific surface area as carbonate‐derived LMR are observed,^[^ ^96^, ^98^, ^99^ ^]^ which possibly stems from the influence of Mn‐rich chemistry on the precursor properties.^[^ ^100^, ^101^ ^]^ At this point, the hydroxide co‐precipitation route for LMR remains less explored. Given that, the single‐crystal approach appears to be the most feasible method to reduce specific surface area, though a method to synthesize single‐crystalline Gen 2 and Gen 3 LMR still needs to be developed.

The surface modification approach includes employing a protective coating layer to lower parasitic reactions of reactive surface oxygen in charged states with the electrolyte (Figure 6b).^[^ ^45^, ^88^, ^102^, ^103^ ^]^ Nevertheless, the highly porous LMR secondary particles,^[^ ^5^, ^104^, ^105^ ^]^ with a specific surface area that can be > 15x of NCM 811 (4.35 vs 0.23 m^2^ g^−1^),^[^ ^88^, ^106^ ^]^ is challenging for a full coating coverage. For example, infusive Co*_x_* B or Li_2_TiO_3_ coatings (Figure 6b) require a sensitive wet chemical or a molten salt method.^[^ ^45^, ^88^, ^102^ ^]^ Alternatively, LMR surface treatments require high‐temperature gas‐solid reactions involving CO_2_, sulfur, or NH_3_ gas.^[^ ^107^, ^108^, ^109^ ^]^ Considering that bulk LMR modification can be more effective than Al_2_O_3_ coating in preventing rollover failure^[^ ^63^ ^]^ and that electrolyte additive can enable 3x longer cycle life than Al_2_O_3_ coating for high‐voltage NCM || graphite cells (4.4 V UCV),^[^ ^110^, ^111^ ^]^ electrochemical performance of coated LMR should be compared with the effect of electrolyte additives to assess the benefit‐to‐effort ratio.

With regards to electrolyte engineering, investigations of more than 300 different electrolyte additive blends suggest that the combination of 2 wt.% prop‐1‐ene‐1,3‐sultone (PES), 1 wt.% ethylene sulfate/1,3,2‐dioxathiolan‐2‐oxide (DTD), and 1 wt.% tris(‐trimethyl‐silyl)‐phosphite (TTSPi), referred to as PES‐211D, is the best additive blend for NCM || graphite Li ion cells operated with EC‐based electrolytes,^[^ ^113^, ^114^, ^115^, ^116^ ^]^ including for high‐voltage operations.^[^ ^110^, ^114^, ^115^ ^]^ With the distinct impact of each additive on the cathode electrolyte interphase (CEI),^[^ ^117^ ^]^ SEI, and the decomposition reaction within the electrolyte,^[^ ^118^, ^119^, ^120^, ^121^, ^122^, ^123^, ^124^ ^]^ their combination in the form of PES‐211D produces only a small amount of gas after high‐temperature storage and cycling experiments and inhibits the growth of cell resistance.^[^ ^113^, ^118^ ^]^

Interestingly, for LMR || graphite cells, the PES‐211D additive combination is outperformed by the 2 wt.% fluoroethylene carbonate (FEC) + 1 wt% lithium difluorophosphate (LiDFP) combination.^[^ ^125^ ^]^ Compared to NCM, LMR is more challenging due to its reactive oxidized oxygen species and/or larger surface area.^[^ ^11^, ^17^, ^58^, ^106^, ^126^ ^]^ Here, FEC acts as an SEI‐forming additive,^[^ ^112^, ^127^ ^]^ while LiDFP (and its decomposition products) suppresses electrode crosstalk by scavenging dissolved *TM*s Figure 6a,c.^[^ ^89^, ^112^ ^]^ Both FEC and LiDFP are more oxidatively stable than vinylene carbonate (VC),^[^ ^112^ ^]^ which appears to be important in LMR || graphite cells.^[^ ^125^ ^]^ VC in particular has a double bond in its ring structure (similar to PES), making it prone to polymerization under oxidative conditions, which is associated with faster capacity fade.^[^ ^126^ ^]^

Considering the tendency of ethylene carbonate (EC) to be chemically oxidized by the reactive surface oxygen of NCMs,^[^ ^103^, ^128^, ^129^ ^]^ further enhancement of capacity retention can be undertaken by eliminating EC to suppress electrolyte oxidation and *TM* dissolution,^[^ ^128^, ^130^ ^]^ which needs to be followed by adding a small amount of SEI‐forming additive, for example, FEC.^[^ ^131^ ^]^ Elaborative electrolyte modifications, e.g., via fluorinating linear carbonates and/or utillizing alternative salts for increased anodic stability,^[^ ^132^, ^133^, ^134^, ^135^ ^]^ is likely redundant when the UCV is only moderately high (see Section 6). This is because fluorination increases cost, toxicity, and storage instability,^[^ ^136^ ^]^ and employing alternative salts does not solve the *TM* dissolution issue,^[^ ^137^ ^]^ is UCV‐limited to 4.4 V,^[^ ^138^ ^]^ or requires fluorinated solvents.^[^ ^133^ ^]^ In contrast, bulk LMR modification that stabilizes lattice oxygen can already prevent rollover failure in practical LMR || graphite cells operated to a UCV of 4.55 V without any electrolyte modifications such as fluorinated solvents.^[^ ^63^ ^]^

## 6 LMR || Graphite Full‐Cell Investigation and Related Charging Protocols

**Figure 7:**
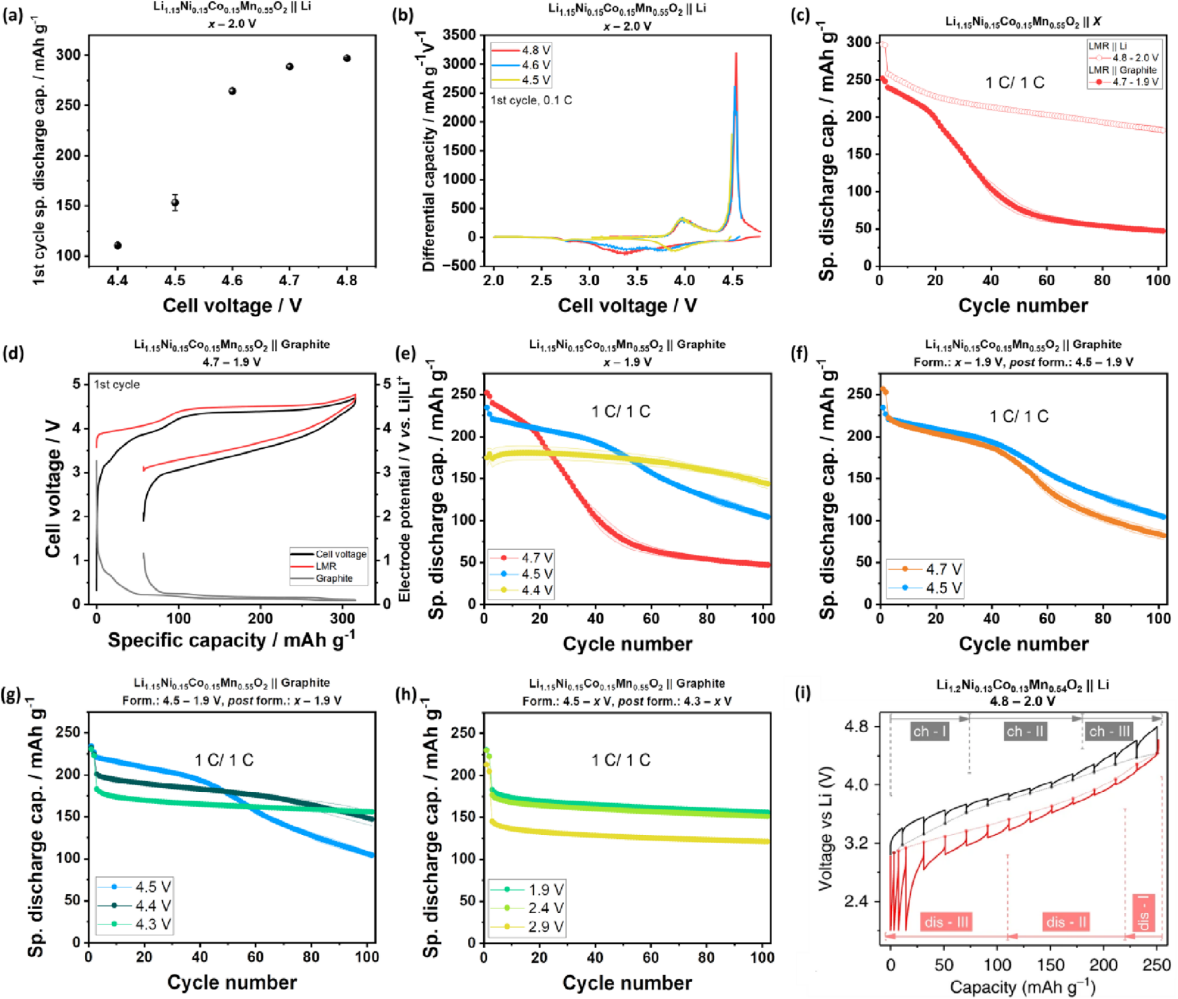
First cycle a) discharge capacity and b) differential capacity versus voltage plots of LMR || Li cells.^[^ ^17^ ^]^ Specific discharge capacity versus cycle number plots of LMR || Li and LMR || graphite cells. d) First‐cycle cell voltage and electrode potential profiles of an LMR || graphite cell with a UCV of 4.7 V. Specific discharge capacity versus cycle number plots of LMR || graphite cells with varied e) UCV, f) formation (form.) cycle UCV, g) *post* formation cycles UCV, h) LCV (4.3 V *post* formation UCV), and i) LCV (4.5 V *post* formation UCV).^[^ ^17^ ^]^ i) LMR || Li voltage profile obtained via the galvanostatic intermittent titration technique.^[^ ^139^ ^]^(a–h) Reproduced with permission.^[^ ^17^ ^]^ Copyright 2024, Wiley‐VCH GmbH. i) Reproduced with permission.^[^ ^139^ ^]^Copyright 2017, Springer Nature.

As seen in **Figure** **7**a, charging LMR || Li cells to UCV > 4.5 V is necessary to obtain high capacity from oxygen redox (Figure 7b).^[^ ^17^, ^139^ ^]^ In more detail, Figure 7b reveals that with a UCV of 4.5 V, the charging process is abruptly stopped when the oxygen oxidation peak has not been reached, limiting the discharge capacity related to oxygen redox in the following discharge step.^[^ ^17^, ^139^ ^]^ Charging to 4.6 V pronouncedly increases discharge capacity since the charging process stops after the oxygen oxidation peak, and further UCV increase only incrementally enhances discharge capacity from the additional oxidation reaction.

Figure 7c shows that LMR || graphite cells with an UCV of 4.7 V (LMR potential ≈ 4.8 V versus Li|Li^+^) have a higher first‐cycle capacity loss (≈ 80% CE) compared to LMR || Li cells (CE ≈ 94%),^[^ ^17^, ^107^ ^]^ due to active lithium loss on graphite (Figure 7d).^[^ ^17^, ^81^ ^]^ Also, a stronger capacity fade (rollover failure) is observed,^[^ ^17^, ^18^, ^85^ ^]^ which is associated with graphite deterioration over the course of electrode crosstalk, i.e.*, TM* dissolution from the *cathode* and their deposition on the *anode* that triggers HSAL in different morphologies, e.g., Li dendrites, which results in rapid lithium inventory losses.^[^ ^17^, ^18^, ^19^, ^85^ ^]^ Potential measures for electrode crosstalk can be for example, surface coatings and/or microstructure modification.^[^ ^45^, ^88^, ^92^, ^140^ ^]^ However, the relevance of electrode crosstalk is often underestimated for LMR due to the frequent use LMR || Li cells in R&D.^[^ ^4^ ^]^ This necessitates comprehensive validations of LMR modification in LMR || graphite full cells without Li reservoir.^[^ ^63^ ^]^ Equally important is the evaluation of gassing behavior, as this can be the limiting factor for long‐term operation.^[^ ^16^ ^]^

While an UCV increase from 4.5 to 4.7 V incrementally enhances discharge capacity, the cycle life greatly decreases (Figure 7e), and a large volume of gas is continuously produced throughout cycling.^[^ ^17^, ^125^ ^]^ Hence, a reasonable UCV of ≈ 4.5 V (LMR potential ≈ 4.6 V versus Li|Li^+^) is proposed,^[^ ^17^ ^]^ as a compromise of capacity and cycle life. At this UCV, the risk of rollover failure can be prevented by *TM*‐scavenging LiDFP additive.^[^ ^141^ ^]^ Further reduction of the UCV is likely problematic as the capacity reduction is large and the increasing capacity throughout cycling poses challenges for capacity balancing (Figure 7e).^[^ ^125^ ^]^ Although a high N/P ratio would prevent lithium plating caused by the increasing capacity, the cell energy density will be compromised.

Setting the formation UCV higher (e.g., 4.7 V) than the *post* formation UCV (e.g., 4.5 V), frequently reported in previous studies,^[^ ^87^, ^98^, ^125^, ^142^ ^]^ appears unnecessary as this can be even detrimental for cycle life (Figure 7f). Moreover, energy density would be compromised as more graphite is needed to accommodate the high capacity in the initial cycles.^[^ ^81^ ^]^ For further cycle life improvement, the *post* formation UCV can be lowered to 4.3 V (Figure 7 g), which results in LMR with a material‐level specific energy between NCM and LFP.^[^ ^17^ ^]^ Although this approach is detrimental for energy density as previously explained, the higher charge capacity during formation cycles can be suitable with low CE anodes, for example, Si‐based anodes. Interestingly, while lower UCV cycling is not beneficial for energy density and cost, an UCV decrease to 3.95 V after high voltage cycling to 4.55 V, can be beneficial to partially restore structural disorder and increase the average discharge voltage.^[^ ^143^ ^]^

Generally, the UCV should be selected according to the inflection point after the oxide oxidation peak in the differential capacity versus voltage plot (Figure 7b), which is influenced by LMR composition. The adjustment of the Ni to Co ratio, in particular, vertically shifts the position of the oxygen oxidation plateau, thus horizontally shifting its position in the differential capacity versus voltage plot.^[^ ^6^, ^14^, ^51^ ^]^ As such, the downshift of the oxygen oxidation peak, due to the more facile oxygen oxidation in Co‐containing LMR,^[^ ^17^, ^51^, ^125^ ^]^ may enable optimal operation of Co‐containing LMR at lower UCVs compared to Co‐free LMR.

With respect to the lower cut‐off voltage (LCV), higher LCVs are generally desired to increase the average discharge voltage.^[^ ^60^, ^144^ ^]^ Nevertheless, the shift of the LMR redox couple throughout cycling, i.e., toward low‐voltage Mn^3+^|Mn^4+^ and Co^2+^|Co^3+^ redox,^[^ ^12^ ^]^ notably limits the accessible discharge capacity if the LCV is not low enough, thus lowering energy density.^[^ ^17^, ^125^ ^]^ For LMR cells with suppressed redox couple shift, e.g., with lower *post* formation UCV (Figure 7g) or with O2‐type LMR,^[^ ^15^, ^17^, ^62^, ^71^ ^]^ the LCV should be set to ≈ 2.5 V as the discharge capacity and specific energy reduction are marginal (Figure 7h).^[^ ^17^, ^125^ ^]^ Furthermore, the highly sluggish discharge process at low SoC regions, which is detrimental to power delivery, can be avoided (Figure 7i).^[^ ^16^, ^60^, ^144^ ^]^

## 7 Comparison to SOTA Cell Chemistry

**Figure 8:**
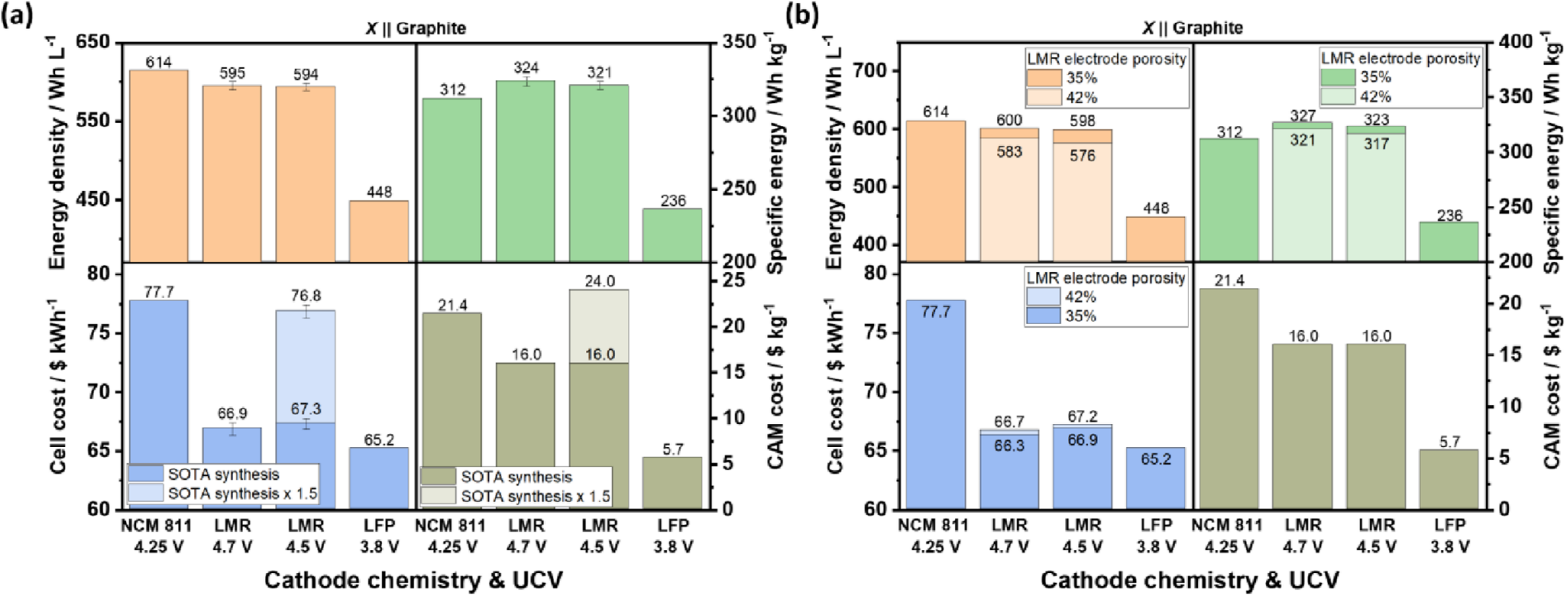
a) Techno‐economic analysis of LMR || graphite cells with UCVs of 4.7 and 4.5 V, compared with NCM 811 || graphite and LFP || graphite cells, and b) the impact of LMR electrode porosities on cell energy density, specific energy, and cost. The error bars in (a) represent the different calculation values due to the different OCV input values.

**Figure** **8**a compares the cell energy density, specific energy, and costs of LMR‐, NCM 811‐, and LFP‐based LIBs. The error bars for LMR‐based cells represent the maximum and minimum calculated values due to the varying voltage values for the modeling, i.e., from actual LMR || graphite cells and from LMR || Li cells subtracted by 0.1 V (Table S3, Supporting Information).^[^ ^17^, ^54^ ^]^ LMR cells with Li_1.15_Mn_0.55_Ni_0.15_Co_0.15_O_2_ from Ningbo Institute of Materials Technology, having first‐cycle CEs > 94% in LMR || Li cells with an UCV of 4.8 V (Table S2, Supporting Information),^[^ ^17^, ^107^ ^]^ are estimated to deliver higher specific energy and slightly lower energy density than the SOTA NCM 811 cell, with cell costs closer to LFP cells.

The lower energy densities of LMR cells result from the higher porosity of LMR secondary particles and the lower crystallographic density of LMR.^[^ ^106^ ^]^ The values for NCM 811 in Figure 8a are calculated with a porosity of 25%, the default BatPaC value for energy‐optimized cells. On the other hand, the LMR electrode porosity is adjusted to 35% (Table S1, Supporting Information), though calendaring to this value is shown to be challenging as it is accompanied by cracking of secondary particles.^[^ ^106^ ^]^ When the LMR electrode is calendered to a recommended porosity of 42%,^[^ ^106^ ^]^ energy density and specific energy decrease, and cell costs slightly increase (Figure 8b). This highlights the importance of reducing the internal pores, i.e., lowering surface area, in LMR secondary particles to increase tap density via novel synthesis methods,^[^ ^63^, ^92^, ^145^ ^]^ which is also argued to improve first‐cycle CE and extend cycle life.^[^ ^58^, ^92^, ^146^ ^]^

Figure 8a shows an only slight decrease in energy density and specific energy when the UCV of the LMR cell is reduced from 4.7 to 4.5 V, while additionally increasing the cycle life (see Section 6), thus a practically pragmatic optimization measure.^[^ ^141^ ^]^ Considering that modified and elaborative synthesis steps, for example, single crystal and/or surface modification, are needed to produce Gen 1 LMRs,^[^ ^92^, ^107^ ^]^ and an ion exchange step is needed to produce Gen 2 and Gen 3 (Table 2),^[^ ^62^, ^71^ ^]^ CAM processing cost increase is expected. In contrast to the weak influence of electrolyte cost on cell cost (Figure S2, Supporting Information), the impact of CAM cost is much stronger. If the proposed CAM modification strategy drastically increases the processing and subsequently the CAM cost by 50% (see SOTA synthesis x 1.5 in Figure 8a), LMR would not be a cost‐competitive chemistry anymore. As such, employing low‐cost LMR with a simple synthesis method should be the focus of LMR R&D.

**Figure 9:**
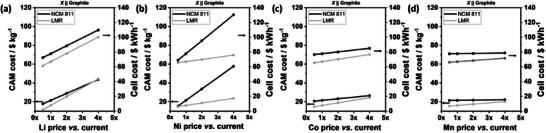
Sensitivity analysis of NCM 811 || graphite and LMR || graphite cells with UCVs of 4.25 and 4.5 V, respectively. The impact of Li, Ni, Co, and Mn price changes with respect to the current market price on the CAM and cell costs is shown in (a), (b), (c), and (d), respectively. For this analysis, the LMR composition is Li_1.15_Mn_0.55_Ni_0.15_Co_0.15_O_2._ Note that the comparison result can be different depending on the LMR composition being evaluated.

Given the Li‐rich composition, LMR CAM (Li_1.15_Mn_0.55_Ni_0.15_Co_0.15_O_2_) is also more sensitive to Li price fluctuation compared to NCM 811 (**Figure** **9**a; Table S10, Supporting Information). Interestingly, although LMR CAM will get more expensive than NCM 811 CAM when the Li price increases to ≈ 4x the current price, the LMR cell cost will remain lower than that of NCM 811 (Figure 9a; Table S10, Supporting Information). It is notable that due to the less amount of CAM needed in LMR versus NCM811 cells, the LMR *cell* cost does not increase as steeply as the LMR *CAM* cost, allowing the LMR cell to keep its cost advantage even at increased Li prices. With regards to Ni price fluctuation, the low Ni content in LMR offers a greater cost advantage for LMR compared to NCM 811 (Figure 9b; Table S11, Supporting Information). While a quadrupling of the Ni price increases the NCM 811 cell cost by 68%, the cost increase for the LMR cell is only 13% (Figure 9b; Table S10, Supporting Information). Concerning Co and Mn price increase, the larger proportion of Co and Mn in LMR versus NCM 811 also affects LMR CAM cost more strongly (Figure 9c,d; Tables S12 and S13, Supporting Information), though the LMR cell will still cost less within the 4x price increase scenario, similar to the case of Li.

## 8 Summary and Outlook

Li/Mn‐rich layered oxide (LMR) cathode active materials (CAM) offer a high practical specific discharge capacity at a still moderately discharge voltage, rendering them promising CAM candidates for high‐energy and low‐cost Li ion batteries (LIBs). However, oxygen redox requires operation to high upper cutoff voltages (UCVs), which causes severe bulk degradation coupled with surface reactivity. Notably, the latter strongly correlates with transition metal (*TM*) dissolution, which relevantly contributes to capacity fade over the course of “electrode crosstalk”, i.e., *TM* deposition on the anode causing high surface area lithium plating and subsequently losses in active lithium inventory.

**Figure 10:**
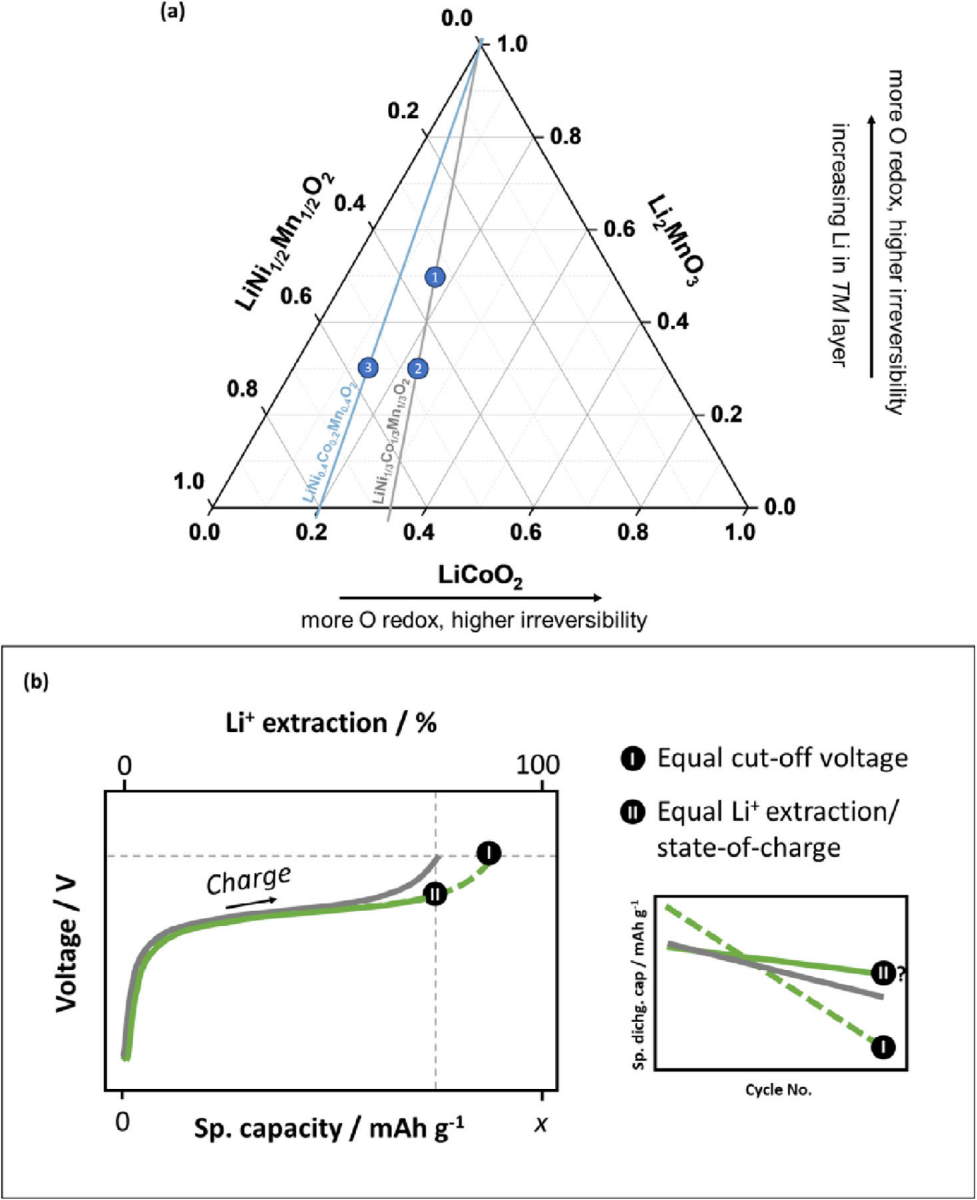
a) Ternary phase diagram of the Li_2_MnO_3_ – LiNi_0.5_Mn_0.5_O_2_ – LiCoO_2_ system for LMR composition design with Ni in 2+ oxidation state: 1) 0.5 Li_2_MnO_3_ · 0.5 LiNi_0.33_Co_0.33_Mn_0.33_O_2_ (Li_1.2_Ni_0.13_Co_0.13_Mn_0.54_O_2_), 2) 0.3 Li_2_MnO_3_ · 0.7 LiNi_0.33_Co_0.33_Mn_0.33_O_2_ (Li_1.13_Ni_0.231_Co_0.128_Mn_0.510_O_2_), and 3) 0.3 Li_2_MnO_3_ · 0.7 LiNi_0.4_Co_0.2_Mn_0.4_O_2_ (Li_1.13_Ni_0.243_Co_0.122_Mn_0.504_O_2_). b) Illustration of voltage versus capacity plots of LMR || Li cells with different LMR compositions and the corresponding capacity versus cycle number plots. The commonly used evaluation procedure to compare different LMR compositions is by setting an equal UCV. As a result, one LMR composition is delithiated more, experiences more stress, and thus typically degrades faster than the other (I). This is typical for LMR with high Li_2_MnO_3_ content,^[^ ^52^ ^]^ low Ni oxidation state,^[^ ^53^ ^]^ and Co‐containing LMR.^[^ ^51^ ^]^ For a more comprehensive comparison of LMR compositions, an additional evaluation procedure with an equal delithiation degree is recommended (II).

Given the bulk instability, LMR design should focus on improving structural stability via composition optimization and novel crystal design. LMR compositions with lower lithium in *TM*‐layer (Li_2_MnO_3_) and lower Co content are likely ideal for high‐energy, low‐cost, and long‐live LMR || graphite cells. In this regard, a systematic study of LMR compositional design based on the LMR ternary phase diagram (**Figure** **10**a) is crucial. Starting from the most common LMR composition 0.5 Li_2_MnO_3_ · 0.5 LiNi_0.33_Co_0.33_Mn_0.33_O_2_ (Li_1.2_Ni_0.13_Co_0.13_Mn_0.54_O_2_; position (1)) as an example, the Li_2_MnO_3_ content can be reduced to produce 0.3 Li_2_MnO_3_ · 0.7 LiNi_0.33_Co_0.33_Mn_0.33_O_2_ (Li_1.13_Ni_0.231_Co_0.128_Mn_0.510_O_2_; position (2)), and then the LiCoO_2_ content can be lowered to form 0.3 Li_2_MnO_3_ · 0.7 LiNi_0.4_Co_0.2_Mn_0.4_O_2_ (Li_1.13_Ni_0.243_Co_0.122_Mn_0.504_O_2_; position (3)). Going beyond the phase diagram, the Co to Ni ratio can then be lowered from 0.5 to 0.2 to form Li_1.13_Ni_0.304_Co_0.061_Mn_0.504_O_2_ to increase the Ni oxidation state from 2+ to 2.2+ for more redox buffering effects. For a more comprehensive evaluation of different LMR chemistries, an evaluation protocol with equal state‐of‐charge (SoC) conditions should be implemented (Figure 10b). With this procedure, high‐capacity LMR compositions (e.g., high Li_2_MnO_3_), which are often argued to suffer from higher irreversibility than low‐capacity LMR compositions (e.g., low Li_2_MnO_3_), may turn out to be structurally more stable when less Li^+^ is extracted from the crystal (Figure 10b, point I → II).

Ideally, Gen 2 and Gen 3 LMRs with O2‐type layered structure should be employed to further enhance structural stability, though issues related to synthesis complexity and costs would limit their practical implementation. For Gen 1 LMR with O3‐type layered structure, cost‐effective microstructure and surface modification strategies should be implemented to deal with the reactive LMR surface. Furthermore, the electrolyte needs to be engineered, with a different approach than electrolyte modification for SOTA LIBs at conventional conditions, to deal with electrode crosstalk phenomena, e.g., via adding lithium difluorophosphate as a *TM* scavenging additive.

For the operation of LMR || graphite cells, it is recommended that the cell UCV is set to ≈ 4.5 V, according to the composition‐dependent lattice oxygen oxidation behavior, to enable an optimal compromise between high capacity and long cycle life. At this moderately high UCV, LMR in LMR || graphite can undergo sufficient oxygen redox activation, and with coating and modified electrolytes, cycling stability can be further improved. Concerning the lower cut‐off voltage, ≈ 2.5 V should be chosen to avoid the highly resistive low SoC region of LMR without a large compromise in capacity.

Techno‐economic analyses suggest that LMR || graphite cells may deliver energy density and specific energy that is comparable to NCM 811 || graphite cells, with cell costs that are closer to LiFePO_4_ || graphite cells. Given the need for bulk, microstructure, and electrolyte modification for long‐term cycling stability, low‐cost modification strategies are required to ensure cost‐competitive LMR || graphite cells. This would be particularly challenging for Gen 2 and Gen 3 LMR, due to the expensive ion‐exchange step in their synthesis process. Finally, metal price sensitivity analyses suggest that LMR cells can still be more cost‐competitive than NCM 811 || graphite cells even when the price of Li, Ni, Co, or Mn quadruples.

## Conflict of Interest

The authors declare no conflict of interest.

## Supporting information



Supporting Information
