# Supplementary material for: Toward Practical Li‐Ion Cells With Li/Mn‐Rich Layered Oxide Cathodes: A Techno‐Economic Perspective on Material and Cell Design
Source: Adv Sci (Weinh). 2025 Sep 30;13(2):e12467. doi: 10.1002/advs.202512467 (PMC12786379; doi:10.1002/advs.202512467)
Supplement: Supplementary file 1 — Supporting Information [file ADVS-13-e12467-s001.docx]

**Supporting Information**

**Towards Practical Li-ion Cells with Li/Mn-rich Layered Oxide Cathodes: A Techno-Economic Perspective on Material and Cell Design**

**Supplementary Note 1 - Background information**

- The BatPaC software used in this study is BatPaC 5.0 from Argonne National Laboratory.^[1]^
- The BatPaC model calculates the mass, size, and cost of a battery pack consisting of modules, made up of stiff pouch cells. In this study, the battery pack size is set to 100 kWh for EV applications. The battery pack is designed to contain 20 modules with 20 pouch cells per module. The plant size is set to 50 GWh, producing 500,000 battery packs per year. Based on the user input, *i.e.*, cell chemistry and electrochemical values, the model adjusts the cell, module, and pack dimensions to obtain a battery pack with the required size.
- The BatPaC model does not consider the impact of cell degradation, safety, and Coulombic/voltage/energy efficiency for cost estimation.
- The energy density and cost modeling for LMR || graphite cells is based on the cell design parameters listed for NCM 811 || graphite (energy) with some adjustments listed in **Table S1.**
- The “N/P capacity ratio after formation” values listed in the Chem worksheet in the BatPaC software are used to estimate the negative electrode loading based on the reversible capacity of the positive and negative electrodes. The set value of 1.1 is not problematic when the assumed ≈ 5% irreversible loss in the positive electrode is consumed for a similar ≈ 5% irreversible loss for SEI formation in the negative electrode. Nevertheless, some LMR compositions modeled in this study (**Table 1**) have much higher irreversible losses compared to graphite, which requires an increase in N/P ratio to avoid lithium plating during formation and to account for residual lithium in graphite that cannot be intercalated back to LMR due to their high irreversibility. The N/P ratios are adjusted to achieve an actual N/P ratio after formation of 1.1, summarized in **Table S2**.
- The input values for LMR || graphite cell open circuit voltage (OCV) at different states of charge (SoC) are estimated by subtracting the OCV values of LMR || Li cells with various LMR compositions by 0.1 V, unless otherwise stated.^[2]^ The accuracy of this approach is illustrated in **Figure 8 (a)**, where the difference in calculation results based on estimated and experimental OCV values are relatively low. For energy density, specific energy, and cell cost calculations, the difference are ≈ 5 Wh L^-1^, ≈ 3 Wh kg^-1^, and ≈ 0.5 $ kWh^-1^, respectively. The estimated OCV values of LMR || graphite cells are listed in **Table S3**, **Table S4** and **Table S5**. The OCV values of LMR || Li cells with various LMR compositions are extracted from the voltage profiles in the respective references.
- For the modeling of each cell chemistries, the positive electrode coating thicknesses are adjusted according to the reversible discharge capacity, to reach an areal capacity of 4.5 mAh cm^-2^ (**Table S2**), similar to that of the Tesla 4680 cell.^[3]^
- The LMR cathode active material (CAM) cost is estimated based on the raw material price (metal basis) for each LMR composition, multiplied by a fixed processing factor. The processing factor (1.43) is calculated based on the price of NCM 811 powder (**Table S6**) divided by the raw material price of NCM 811 based on the precursor price listed in **Table S7**.^[4]^ It is notable that this approach may result in calculation inaccuracy when comparing NCM 811 and LMR due to the difference in the processing factor of these materials. Furthermore, different synthesis parameters for different LMR compositions may also alter the processing factor. Thus, the cell cost values presented in this work should only be considered as relative comparison to help identify the most cost-competitive LMR chemistry.
- The values estimated in this study assume no changes in electrolyte cost for LMR, compared to SOTA LIBs. As illustrated in **Figure S2**, a 50% increase in LMR electrolyte cost increases cell cost by 1.4 $ kWh^-1^, which is still far lower than the NCM 811 cell cost. In contrast, a 50% increase in CAM cost increases cell cost by 9.5 $ kWh^-1^. This shows the relatively weak influence of electroyte cost on cell cost.

**Table S1**. List of adjusted input parameters in the “Chem” worksheet for LMR || graphite cells

| **Parameter** | **NCM 811** | **LMR** | **Remarks for LMR** | **Reference** |
| --- | --- | --- | --- | --- |
| Density, g/cm^3^ | 4.65 | 4.35 |  | ^[5]^ |
| Void, vol% of positive electrode | 25 | 35/42 | Higher porosity due to internal pores | ^[5]^ |
| Active material particle radius, µm | 6 | 6 |  | ^[6]^ |
| Area specific impedance at 2-sec burst, T0 = 25 °C, ohm-cm² | | | Estimated to be 50% higher than NCA | ^[7]^ |
| ASI at 2-sec burst, 0% SOC, ohm-cm² | 30.56 | 45.75 |  |  |
| ASI at 2-sec burst, 10% SOC, ohm-cm² | 17.98 | 26.91 |  |  |
| ASI at 2-sec burst, 20% SOC, ohm-cm² | 11.98 | 17.94 |  |  |
| ASI at 2-sec burst, 30% SOC, ohm-cm² | 11.09 | 16.59 |  |  |
| ASI at 2-sec burst, 40% SOC, ohm-cm² | 9.91 | 14.83 |  |  |
| ASI at 2-sec burst, 50% SOC, ohm-cm² | 10.11 | 15.13 |  |  |
| ASI at 2-sec burst, 60% SOC, ohm-cm² | 10.49 | 15.70 |  |  |
| ASI at 2-sec burst, 70% SOC, ohm-cm² | 10.86 | 16.26 |  |  |
| ASI at 2-sec burst, 80% SOC, ohm-cm² | 10.91 | 16.33 |  |  |
| ASI at 2-sec burst, 90% SOC, ohm-cm² | 10.99 | 16.46 |  |  |
| ASI at 2-sec burst, 100% SOC, ohm-cm² | 11.54 | 17.28 |  |  |
| Area specific impedance at 10-sec burst, T0 = 25 °C, ohm-cm² | | |  |  |
| ASI at 10-sec burst, 0% SOC, ohm-cm² | 47.33 | 70.91 |  |  |
| ASI at 10-sec burst, 10% SOC, ohm-cm² | 27.84 | 41.71 |  |  |
| ASI at 10-sec burst, 20% SOC, ohm-cm² | 18.56 | 27.81 |  |  |
| ASI at 10-sec burst, 30% SOC, ohm-cm² | 16.03 | 24.01 |  |  |
| ASI at 10-sec burst, 40% SOC, ohm-cm² | 14.17 | 21.23 |  |  |
| ASI at 10-sec burst, 50% SOC, ohm-cm² | 14.59 | 21.85 |  |  |
| ASI at 10-sec burst, 60% SOC, ohm-cm² | 14.85 | 22.25 |  |  |
| ASI at 10-sec burst, 70% SOC, ohm-cm² | 15.33 | 22.97 |  |  |
| ASI at 10-sec burst, 80% SOC, ohm-cm² | 15.46 | 23.16 |  |  |
| ASI at 10-sec burst, 90% SOC, ohm-cm² | 15.91 | 23.84 |  |  |
| ASI at 10-sec burst, 100% SOC, ohm-cm² | 16.71 | 25.03 |  |  |

**Table S2**. List of N/P ratio input, coating thickness, and CAM costs for various LMR chemistries. Note that for data presented in Figure 2, 5, and Figure S1, an unadjusted BatPac N/P ratio input of 1.1 results in a *post* formation N/P ratio >1.10 due the low CE of LMR. This requires BatPac N/P ratio input adjustments to enable a *post* formation N/P ratio of 1.1. The modeling of Li_2_MnO_3_ content in Figure 2 (a) is based on the LMR composition *x* Li_2_MnO_3_∙ (1˗*x*) LiNi_0.38_Co_0.21_Mn_0.41_O_2_.^[5,7]^

| **Figure** | **Material** | **Ref.** | **BatPac N/P ratio input** | **(+) coating thickness / μm** | **Sp. dischg. cap. in Li cells / mAh g^-1^** | **(+) areal dischrge cap. / mAh cm^-2^** | **CE / %** | **(+) areal charge cap. / mAh cm^-2^** | **(+) Li loss / mAh cm^-2^** | **(-) areal cap. / mAh cm^-2^** | **(-) form. losses / mAh cm^-2^** | **Remaining (-) areal cap. / mAh cm^-2^** | **Li inventory / mAh cm^-2^** | **N/P ratio *post* form.** | **CAM cost / $ kg^-1^** |
| --- | --- | --- | --- | --- | --- | --- | --- | --- | --- | --- | --- | --- | --- | --- | --- |
| 8 (a) | Li_1.15_Mn_0.55_Ni_0.15_Co_0.15_O_2_ (4.7 V) | ^[8]^ | 1.1 | 59 | 297 | 4.5 | 94 | 4.79 | 0.29 | 4.95 | 0.28 | 4.94 | 4.51 | 1.10 | 15.98 |
|  | Li_1.15_Mn_0.55_Ni_0.15_Co_0.15_O_2_ (4.5 V) |  | 1.1 | 64 | 275 | 4.5 | 95 | 4.74 | 0.24 | 4.95 | 0.28 | 4.95 | 4.46 | 1.11 |  |
|  | NCM 811 |  | 1.1 | 67 | 214 | 4.5 | 95 | 4.74 | 0.24 | 4.95 | 0.28 | 4.95 | 4.46 | 1.11 | 21.36 |
|  | LFP |  | 1.1 | 120 | 157 | 4.5 | 95 | 4.74 | 0.24 | 4.95 | 0.28 | 4.95 | 4.46 | 1.11 | 5.68 |
| 4 (a) | Li_1.14_*TM*_0.86_O_2_ | ^[5,7,9]^ | 1.32 | 66 | 268 | 4.5 | 85 | 5.29 | 0.79 | 5.94 | 0.33 | 5.48 | 4.96 | 1.10 | 16.37 |
|  | Li_1.17_*TM*_0.83_O_2_ |  | 1.29 | 61 | 287 | 4.5 | 86 | 5.23 | 0.73 | 5.81 | 0.33 | 5.40 | 4.91 | 1.10 | 15.99 |
|  | Li_1.20_*TM*_0.80_O_2_ |  | 1.37 | 62 | 283 | 4.5 | 83 | 5.42 | 0.92 | 6.17 | 0.35 | 5.59 | 5.08 | 1.10 | 15.68 |
| 4 (b) | Li_1.2_Ni_0.2_Mn_0.6_O_2_ | ^[10]^ | 1.42 | 72 | 245 | 4.5 | 81 | 5.56 | 1.06 | 6.39 | 0.36 | 5.69 | 5.20 | 1.10 | 15.10 |
|  | Li_1.2_Ni_0.25_Mn_0.55_O_2_ |  | 1.55 | 81 | 217 | 4.5 | 77 | 5.84 | 1.34 | 6.98 | 0.39 | 6.02 | 5.45 | 1.10 | 15.80 |
|  | Li_1.2_Ni_0.3_Mn_0.5_O_2_ |  | 1.79 | 92 | 192 | 4.5 | 70 | 6.43 | 1.93 | 8.06 | 0.45 | 6.58 | 5.98 | 1.10 | 16.50 |
| S1 (a) | Li_1.2_Ni_0.13_Co_0.13_Mn_0.54_O_2_ | ^[10]^ | 1.51 | 69 | 253 | 4.5 | 78 | 5.77 | 1.27 | 6.80 | 0.38 | 5.91 | 5.39 | 1.10 | 16.07 |
|  | Li_1.2_Ni_0.17_Co_0.13_Mn_0.50_O_2_ |  | 1.58 | 77 | 228 | 4.5 | 76 | 5.92 | 1.42 | 7.11 | 0.40 | 6.09 | 5.52 | 1.10 | 16.63 |
|  | Li_1.2_Ni_0.22_Co_0.13_Mn_0.45_O_2_ |  | 1.71 | 83 | 211 | 4.5 | 72 | 6.25 | 1.75 | 7.70 | 0.43 | 6.38 | 5.82 | 1.10 | 17.33 |
| S1 (b) | Li_1.2_Ni_0.2_Mn_0.6_O_2_ | ^[11]^ | 1.61 | 65 | 270 | 4.5 | 75 | 6.00 | 1.50 | 7.25 | 0.41 | 6.15 | 5.59 | 1.10 | 15.10 |
|  | Li_1.2_Ni_0.3_Mn_0.5_O_2_ |  | 1.71 | 70 | 253 | 4.5 | 72 | 6.25 | 1.75 | 7.70 | 0.43 | 6.38 | 5.82 | 1.10 | 16.50 |
|  | Li_1.2_Ni_0.4_Mn_0.4_O_2_ |  | 1.75 | 73 | 242 | 4.5 | 71 | 6.34 | 1.84 | 7.88 | 0.44 | 6.48 | 5.90 | 1.10 | 17.90 |
| 4 (c) | Li_1.2_Ni_0.2_Mn_0.6_O_2_ | ^[12]^ | 1.51 | 73 | 240 | 4.5 | 78 | 5.77 | 1.27 | 6.80 | 0.38 | 5.91 | 5.39 | 1.10 | 15.10 |
|  | Li_1.2_Ni_0.13_Co_0.13_Mn_0.54_O_2_ |  | 1.4 | 62 | 284 | 4.5 | 82 | 5.49 | 0.99 | 6.30 | 0.35 | 5.67 | 5.13 | 1.10 | 16.07 |
|  | Li_1.2_Co_0.4_Mn_0.4_O_2_ |  | 1.91 | 78 | 226 | 4.5 | 67 | 6.72 | 2.22 | 8.60 | 0.48 | 6.86 | 6.23 | 1.10 | 18.33 |
| 4 (d) | Li_1.13_Ni_0.275_Mn_0.580_O_2_ | ^[13]^ | 1.22 | 66 | 268 | 4.5 | 89 | 5.07 | 0.57 | 5.49 | 0.31 | 5.23 | 4.76 | 1.10 | 15.25 |
|  | Li_1.13_Ni_0.181_Co_0.089_Mn_0.560_O_2_ |  | 1.29 | 62 | 282 | 4.5 | 86 | 5.23 | 0.73 | 5.81 | 0.33 | 5.40 | 4.90 | 1.10 | 15.46 |
|  | Li_1.13_Ni_0.130_Co_0.135_Mn_0.551_O_2_ |  | 1.18 | 61 | 286 | 4.5 | 90 | 4.97 | 0.47 | 5.31 | 0.30 | 5.13 | 4.68 | 1.10 | 15.53 |
|  | Li_1.13_Ni_0.086_Co_0.174_Mn_0.544_O_2_ |  | 1.32 | 64 | 279 | 4.5 | 85 | 5.30 | 0.80 | 5.94 | 0.33 | 5.48 | 4.96 | 1.10 | 15.57 |

**Table S3**. List of estimated LMR || graphite cell voltage with different LMR modifications. The OCV values are calculated by subtracting the LMR || Li cell voltage extracted from the respective references by 0.1 at 10 – 100% SOC.^[2]^ The asterisk (*) indicates that the values are obtained from actual LMR || graphite cells in the respective reference.

| **OCV at  *x*% SOC / V** | **Modification** | | | | | | |
| --- | --- | --- | --- | --- | --- | --- | --- |
|  | **UCV (Li_1.15_Mn_0.55_Ni_0.15_Co_0.15_O_2_)^[8]^ Fig. 8 (a)** | | | | **Li to *TM* ratio**  **Fig. 4 (a)** | | |
|  | **4.7 V*** | **4.7 V** | **4.5 V*** | **4.5 V** | **Li_1.14_ *TM*_0.86_O_2_** | **Li_1.17_ *TM*_0.83_O_2_** | **Li_1.20_ *TM*_0.80_O_2_** |
| 0 | 2.00 | 2.00 | 2.00 | 2.00 | 2.00 | 2.00 | 2.00 |
| 10 | 2.96 | 2.87 | 2.99 | 2.88 | 2.98 | 2.86 | 2.63 |
| 20 | 3.11 | 3.06 | 3.15 | 3.10 | 3.19 | 3.03 | 2.97 |
| 30 | 3.23 | 3.19 | 3.29 | 3.24 | 3.32 | 3.18 | 3.09 |
| 40 | 3.35 | 3.29 | 3.42 | 3.36 | 3.46 | 3.32 | 3.22 |
| 50 | 3.48 | 3.41 | 3.55 | 3.49 | 3.57 | 3.45 | 3.35 |
| 60 | 3.61 | 3.54 | 3.66 | 3.62 | 3.68 | 3.58 | 3.48 |
| 70 | 3.75 | 3.69 | 3.77 | 3.74 | 3.80 | 3.71 | 3.62 |
| 80 | 3.92 | 3.87 | 3.92 | 3.89 | 3.98 | 3.90 | 3.78 |
| 90 | 4.19 | 4.14 | 4.15 | 4.12 | 4.20 | 4.13 | 4.00 |
| 100 | 4.70 | 4.70 | 4.50 | 4.50 | 4.70 | 4.70 | 4.70 |

**Table S4**. List of estimated LMR || graphite cell voltage with different LMR modifications. The OCV values are calculated by subtracting the LMR || Li cell voltage extracted from the respective references by 0.1 at 10 – 100% SOC.^[2]^

| **OCV at  *x*% SOC / V** | **Modification** | | | | | | | | |
| --- | --- | --- | --- | --- | --- | --- | --- | --- | --- |
|  | **Ni to Mn ratio^[10]^**  **Fig. 4 (b)** | | | **Ni to Mn ratio^[10]^**  **Fig. S1(a)** | | | **Ni to Mn ratio^[11]^**  **Fig. S1 (b)** | | |
|  | **Li_1.2_Ni_0.2_ Mn_0.6_O_2_** | **Li_1.2_Ni_0.25_ Mn_0.55_O_2_** | **Li_1.2_Ni_0.3_ Mn_0.5_O_2_** | **Li_1.2_ Ni_0.13_Co_0.13_ Mn_0.54_O_2_** | **Li_1.2_ Ni_0.17_Co_0.13_ Mn_0.50_O_2_** | **Li_1.2_ Ni_0.22_Co_0.13_ Mn_0.45_O_2_** | **Li_1.2_Ni_0.2_ Mn_0.6_O_2_** | **Li_1.2_Ni_0.3_ Mn_0.5_O_2_** | **Li_1.2_Ni_0.4_ Mn_0.4_O_2_** |
| 0 | 2.00 | 2.00 | 2.00 | 2.00 | 2.00 | 2.00 | 2.00 | 2.00 | 2.00 |
| 10 | 2.91 | 3.03 | 3.11 | 3.04 | 3.09 | 3.08 | 2.75 | 2.74 | 2.39 |
| 20 | 3.15 | 3.25 | 3.33 | 3.21 | 3.25 | 3.27 | 3.01 | 3.06 | 3.06 |
| 30 | 3.27 | 3.39 | 3.50 | 3.31 | 3.37 | 3.38 | 3.12 | 3.22 | 3.25 |
| 40 | 3.39 | 3.53 | 3.60 | 3.40 | 3.48 | 3.49 | 3.20 | 3.33 | 3.42 |
| 50 | 3.53 | 3.63 | 3.68 | 3.51 | 3.58 | 3.59 | 3.28 | 3.47 | 3.55 |
| 60 | 3.64 | 3.70 | 3.76 | 3.63 | 3.67 | 3.69 | 3.39 | 3.61 | 3.64 |
| 70 | 3.76 | 3.82 | 3.87 | 3.75 | 3.79 | 3.80 | 3.57 | 3.74 | 3.77 |
| 80 | 3.92 | 3.96 | 4.00 | 3.91 | 3.95 | 3.95 | 3.77 | 3.90 | 3.95 |
| 90 | 4.13 | 4.14 | 4.15 | 4.14 | 4.17 | 4.14 | 4.07 | 4.12 | 4.15 |
| 100 | 4.70 | 4.70 | 4.70 | 4.70 | 4.70 | 4.70 | 4.70 | 4.70 | 4.70 |

**Table S5**. List of estimated LMR || graphite cell voltage with different LMR modifications. The OCV values are calculated by subtracting the LMR || Li cell voltage extracted from the respective references by 0.1 at 10 – 100% SOC.^[2]^

| **OCV at  *x*% SOC / V** | **Modification** | | | | | | |
| --- | --- | --- | --- | --- | --- | --- | --- |
|  | **Ni to Co ratio^[12]^**  **Fig. 4 (c)** | | | **Ni to Co ratio^[13]^**  **Fig. 4 (d)** | | | |
|  | **Li_1.2_ Ni_0.2_ Mn_0.6_O_2_** | **Li_1.2_ Ni_0.13_Co_0.13_ Mn_0.54_O_2_** | **Li_1.2_ Co_0.4_ Mn_0.4_O_2_** | **Li_1.13_ Ni_0.275_ Mn_0.580_O_2_** | **Li_1.13_ Ni_0.181_Co_0.089_ Mn_0.560_O_2_** | **Li_1.13_ Ni_0.130_Co_0.135_ Mn_0.551_O_2_** | **Li_1.13_ Ni_0.086_Co_0.174_ Mn_0.544_O_2_** |
| 0 | 2.00 | 2.00 | 2.00 | 2.00 | 2.00 | 2.00 | 2.00 |
| 10 | 2.85 | 3.01 | 2.90 | 3.00 | 3.01 | 2.85 | 2.84 |
| 20 | 3.10 | 3.17 | 3.12 | 3.19 | 3.17 | 3.06 | 3.05 |
| 30 | 3.22 | 3.26 | 3.22 | 3.36 | 3.29 | 3.18 | 3.17 |
| 40 | 3.35 | 3.34 | 3.30 | 3.50 | 3.42 | 3.29 | 3.27 |
| 50 | 3.50 | 3.44 | 3.39 | 3.62 | 3.54 | 3.40 | 3.37 |
| 60 | 3.62 | 3.56 | 3.51 | 3.70 | 3.65 | 3.53 | 3.49 |
| 70 | 3.74 | 3.70 | 3.69 | 3.81 | 3.76 | 3.66 | 3.64 |
| 80 | 3.91 | 3.86 | 3.86 | 3.99 | 3.92 | 3.84 | 3.82 |
| 90 | 4.12 | 4.13 | 4.06 | 4.19 | 4.16 | 4.08 | 4.04 |
| 100 | 4.70 | 4.70 | 4.70 | 4.70 | 4.70 | 4.70 | 4.70 |

**Table S6**. Cell component price as of July 2024.

| **Cell component** | **Price / $ kg^-1^** | **Reference** |
| --- | --- | --- |
| NCM622 powder | 16.88 | <https://www.metal.com/Lithium%20Battery%20Cathode%20Precursor%20and%20Material/201805220001> |
| NCM811 powder | 21.36 | <https://www.metal.com/Lithium%20Battery%20Cathode%20Precursor%20and%20Material/202006100012> |
| LFP powder | 5.68 | <https://www.metal.com/Lithium%20Battery%20Cathode%20Precursor%20and%20Material/202406250014> |
| Electrolyte | 6.3 | <https://www.metal.com/Electrolyte/202006100003> |

**Table S7**. Raw material price as of July 2024.

| **Raw material** | **Price / $ kg^-1^** | **Reference** | **Metal content / wt.%** | **Precursor price / $ kg_metal_^-1^** | **Precursor price / $ mol_metal_^-1^** |
| --- | --- | --- | --- | --- | --- |
| NiSO_4_·6H_2_O | 3.92 | <https://www.metal.com/Nickel/201908270001> | 22 | 17.56 | 1.03 |
| CoSO_4_·7H_2_O | 4.05 | <https://www.metal.com/Chemical-Compound/201102250381> | 21 | 19.32 | 1.14 |
| MnSO_4_·H_2_O | 0.88 | <https://www.metal.com/Manganese/201805300001> | 33 | 2.71 | 0.15 |
| LiOH·H_2_O | 12.01 | <https://www.metal.com/Lithium/202106020003> | 17 | 72.61 | 0.50 |
| Li_2_CO_3_ | 12.08 | <https://www.metal.com/Chemical-Compound/201102250059> | 19 | 64.31 | 0.45 |

**Table S8**. Comparison of cation content per formula and Ni ion charge of LMR compositions with varied Co/Ni ratios from a previous report.^[13]^ Note that with the proposed composition, the cation content (Li + *TM*) in the Li*TM*O_2_ is < 2, implying that there are some vacancies in the crystal structure. Ni ion charge is calculated by considering a fixed charge of +1, +4, and +3 for Li, Mn, and, Co ions, respectively. Note that the total cation stoichiometry in these material is not 2, indicating the presence of vacancies.

| **Co/Ni** | **Material** | **Content per formula unit** | | | **Total charge** | | | | **Ni ion charge** |
| --- | --- | --- | --- | --- | --- | --- | --- | --- | --- |
|  |  | **Li** | ***TM*** | **Cation** | **Li** | **Mn** | **Co** | **Ni** |  |
| 0 | Li_1.13_Ni_0.275_Mn_0.580_O_2_ | 1.13 | 0.855 | 1.985 | 1.13 | 2.32 | 0 | 0.55 | 2.00 |
| 0.5 | Li_1.13_Ni_0.181_Co_0.089_Mn_0.560_O_2_ | 1.13 | 0.83 | 1.96 | 1.13 | 2.24 | 0.27 | 0.55 | 2.01 |
| 1 | Li_1.13_Ni_0.130_Co_0.135_Mn_0.551_O_2_ | 1.13 | 0.816 | 1.946 | 1.13 | 2.20 | 0.41 | 0.55 | 2.01 |
| 2 | Li_1.13_Ni_0.086_Co_0.174_Mn_0.544_O_2_ | 1.13 | 0.804 | 1.934 | 1.13 | 2.18 | 0.52 | 0.55 | 2.00 |

**Table S9**. Comparison of cation content per formula and Ni ion charge of LMR compositions with varied Co/Ni ratios. The Co/Ni = 2 composition corresponds to 0.3 Li_2_MnO_3_ • 0.7 LiNi_0.25_Co_0.50_Mn_0.25_O_2_. Note that for this composition, the maximum Co/Ni ratio is 2 to maintain Ni ions in +2 oxidation state, and compositions with lower Co/Ni ratios are obtained based on this composition. For layered oxides with a Ni ion oxidation state of +2, different maximum values for Co/Ni ratios can be obtained from two extreme compositions in the phase diagram, *i.e.,* LiNi_0.5_Mn_0.5_O_2_ and LiCoO_2_.^[14]^ Ni ion charge is calculated by considering a fixed charge of +1, +4, and +3 for Li, Mn, and, Co ions, respectively.

| **Co/Ni** | **Material** | **Content per formula unit** | | | **Total charge** | | | | **Ni ion charge** |
| --- | --- | --- | --- | --- | --- | --- | --- | --- | --- |
|  |  | **Li** | ***TM*** | **Cation** | **Li** | **Mn** | **Co** | **Ni** |  |
| 0 | Li_1.13_Ni_0.457_Mn_0.41_O_2_ | 1.13 | 0.870 | 2.000 | 1.13 | 1.65 | 0 | 1.22 | 2.67 |
| 0.5 | Li_1.13_Ni_0.304_Co_0.152_Mn_0.413_O_2_ | 1.13 | 0.870 | 2.000 | 1.13 | 1.65 | 0.46 | 1.22 | 2.50 |
| 1 | Li_1.13_Ni_0.228_Co_0.228_Mn_0.413_O_2_ | 1.13 | 0.870 | 2.000 | 1.13 | 1.65 | 0.68 | 1.22 | 2.33 |
| 2 | Li_1.13_Ni_0.152_Co_0.304_Mn_0.413_O_2_ | 1.13 | 0.870 | 2.000 | 1.13 | 1.65 | 0.91 | 1.22 | 2.00 |

**Table S10**. NCM 811 and LMR CAM cost changes as a function of Li price changes and their influence on cell cost..

| **Li price *vs.* current** | **CAM cost / $ kg^-1^** | | **Cell cost / $ kWh^-1^** | |
| --- | --- | --- | --- | --- |
|  | **NCM 811** | **LMR** | **NCM 811** | **LMR** |
| 0.5x | 17.67 | 11.28 | 72.4 | 61.3 |
| 1x | 21.36 | 15.98 | 77.7 | 66.9 |
| 2x | 28.75 | 25.39 | 88.4 | 78.0 |
| 4x | 43.52 | 44.21 | 109.8 | 100.2 |

**Table S11**. NCM 811 and LMR CAM cost changes as a function of Mn price changes and their influence on cell cost..

| **Ni price *vs.* current** | **CAM cost / $ kg^-1^** | | **Cell cost / $ kWh^-1^** | |
| --- | --- | --- | --- | --- |
|  | **NCM 811** | **LMR** | **NCM 811** | **LMR** |
| 0.5x | 15.32 | 14.73 | 69.0 | 65.4 |
| 1x | 21.36 | 15.98 | 77.7 | 66.9 |
| 2x | 33.44 | 18.49 | 95.2 | 69.8 |
| 4x | 57.61 | 23.51 | 130.3 | 75.8 |

**Table S12**. NCM 811 and LMR CAM cost changes as a function of Co price changes and their influence on cell cost..

| **Co price *vs.* current** | **CAM cost / $ kg^-1^** | | **Cell cost / $ kWh^-1^** | |
| --- | --- | --- | --- | --- |
|  | **NCM 811** | **LMR** | **NCM 811** | **LMR** |
| 0.5x | 20.53 | 14.61 | 76.5 | 65.2 |
| 1x | 21.36 | 15.98 | 77.7 | 66.9 |
| 2x | 23.03 | 18.72 | 80.1 | 70.1 |
| 4x | 26.37 | 24.19 | 85.0 | 76.6 |

**Table S13**. NCM 811 and LMR CAM cost changes as a function of Mn price changes and their influence on cell cost..

| **Mn price *vs.* current** | **CAM cost / $ kg^-1^** | | **Cell cost / $ kWh^-1^** | |
| --- | --- | --- | --- | --- |
|  | **NCM 811** | **LMR** | **NCM 811** | **LMR** |
| 0.5x | 21.25 | 15.32 | 77.6 | 66.1 |
| 1x | 21.36 | 15.98 | 77.7 | 66.9 |
| 2x | 21.58 | 17.31 | 78.0 | 68.4 |
| 4x | 22.01 | 19.97 | 78.7 | 71.6 |


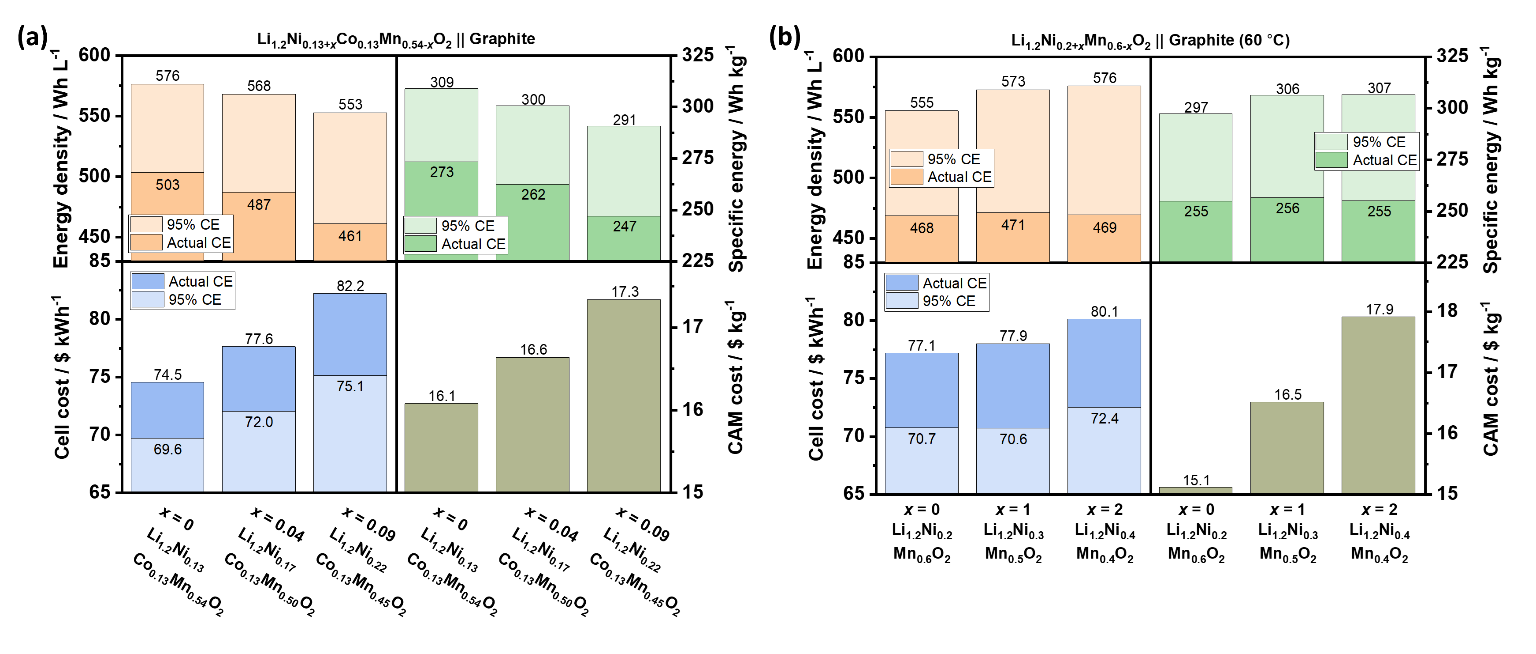


**Figure S1.** Techno-economic analysis of LMR || graphite cells with varied (a) Ni to Mn ratio/Ni oxidation state for Co-doped LMR, and (b) Ni to Mn ratio/Ni oxidation state for LMR operated at 60 °C.

**
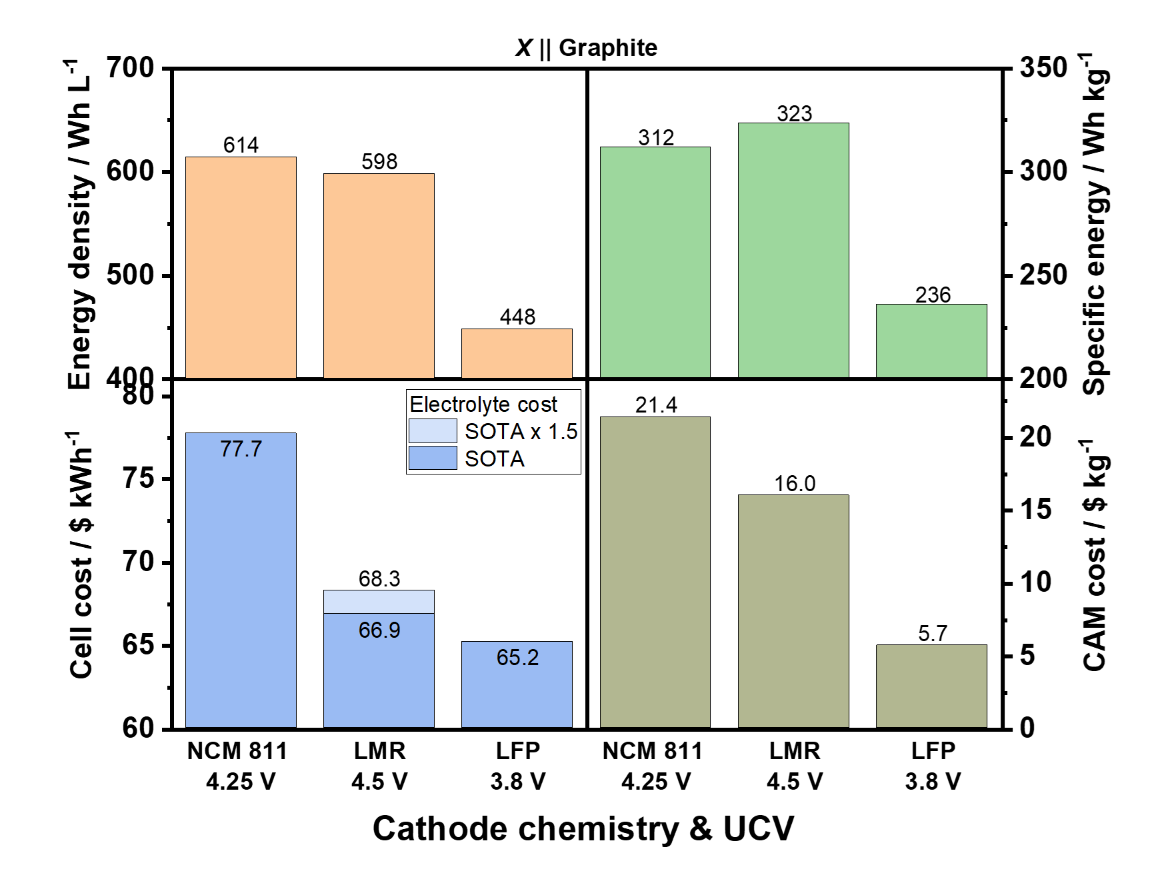
**

**Figure S2.** Techno-economic analysis of LMR || graphite cells with a hypothetical increase of electrolyte cost by 50% (SOTA x 1.5), compared to SOTA cathodes with 25% porosity.

**References**

[1] K. Knehr, J. Kubal, P. Nelson, S. Ahmed, *Battery Performance and Cost Modeling for Electric-Drive Vehicles (A Manual for BatPaC v5.0)* **2022**.

[2] S. Ahmed, S. E. Trask, D. W. Dees, P. A. Nelson, W. Lu, A. R. Dunlop, B. J. Polzin, A. N. Jansen, *J. Power Sources* **2018**, *403*, 56.

[3] M. Ank, A. Sommer, K. Abo Gamra, J. Schöberl, M. Leeb, J. Schachtl, N. Streidel, S. Stock, M. Schreiber, P. Bilfinger, C. Allgäuer, P. Rosner, J. Hagemeister, M. Rößle, R. Daub, M. Lienkamp, *J. Electrochem. Soc.* **2023**, *170*, 120536.

[4] C. Vaalma, D. Buchholz, M. Weil, S. Passerini, *Nat. Rev. Mater.* **2018**, *3*.

[5] D. Schreiner, T. Zünd, F. J. Günter, L. Kraft, B. Stumper, F. Linsenmann, M. Schüßler, R. Wilhelm, A. Jossen, G. Reinhart, H. A. Gasteiger, *J. Electrochem. Soc.* **2021**, *168*, 30507.

[6] M. Yoon, Y. Dong, J. Hwang, J. Sung, H. Cha, K. Ahn, Y. Huang, S. J. Kang, J. Li, J. Cho, *Nat. Energy* **2021**, *6*, 362.

[7] L. Kraft, T. Zünd, D. Schreiner, R. Wilhelm, F. J. Günter, G. Reinhart, H. A. Gasteiger, A. Jossen, *J. Electrochem. Soc.* **2021**, *168*, 20537.

[8] A. Arifiadi, T. Brake, F. Demelash, B. Ying, K. Kleiner, H. Hur, S. Wiemers‐Meyer, M. Winter, J. Kasnatscheew, *Adv. Energy. Sustain. Res.* **2024**.

[9] T. Teufl, B. Strehle, P. Müller, H. A. Gasteiger, M. A. Mendez, *J. Electrochem. Soc.* **2018**, *165*, A2718-A2731.

[10] J. C. Knight, A. Manthiram, *J. Mater. Chem. A* **2015**, *3*, 22199.

[11] K. Ku, J. Hong, H. Kim, H. Park, W. M. Seong, S.-K. Jung, G. Yoon, K.-Y. Park, H. Kim, K. Kang, *Adv. Energy Mater.* **2018**, *8*, 1800606.

[12] B. Li, Z. Zhuo, L. Zhang, A. Iadecola, X. Gao, J. Guo, W. Yang, A. V. Morozov, A. M. Abakumov, J.-M. Tarascon, *Nat. Mater.* **2023**, *22*, 1370.

[13] S. Shen, Y. Hong, F. Zhu, Z. Cao, Y. Li, F. Ke, J. Fan, L. Zhou, L. Wu, P. Dai, M. Cai, L. Huang, Z. Zhou, J. Li, Q. Wu, S. Sun, *ACS Appl. Mater. Interfaces* **2018**, *10*, 12666.

[14] E. Boivin, N. Guerrini, R. A. House, J. G. Lozano, L. Jin, G. J. Rees, J. W. Somerville, C. Kuss, M. R. Roberts, P. G. Bruce, *Adv. Funct. Mater.* **2021**, *31*.
